# A Phase Ia/b study of MEK1/2 inhibitor binimetinib with MET inhibitor crizotinib in patients with *RAS* mutant advanced colorectal cancer (MErCuRIC)

**DOI:** 10.1186/s12885-025-14068-1

**Published:** 2025-04-10

**Authors:** Francesca Aroldi, Elena Elez, Thierry André, Geraldine Perkins, Hans Prenen, Vlad Popovici, Peter Gallagher, Jennifer Houlden, Linda Collins, Corran Roberts, Christian Rolfo, Federica Di Nicolantonio, Margaret Grayson, Ruth Boyd, Karolien Bettens, Jurgen Delfavero, Victoria Coyle, Mark Lawler, Hajrah Khawaja, Pierre Laurent-Puig, Manuel Salto-Tellez, Tim S. Maughan, Josep Tabernero, Richard Adams, Robert Jones, Bryan T. Hennessy, Alberto Bardelli, Marc Peeters, Mark R. Middleton, Richard H. Wilson, Sandra Van Schaeybroeck, on behalf of the MErCuRIC Trial Consortium

**Affiliations:** 1 Department of Oncology, University of Oxford, Old Road Campus Research Building Roosevelt Drive, Oxford, OX3 7DQ UK; 2 Vall d’Hebron University Hospital and Institute of Oncology (VHIO), 08035 Barcelona, Spain; 3 Department of Medical Oncology, Sorbonne Université, Hôpital Saint Antoine, 75012 Paris, France; 4 Department of GI Oncology, Hôpital Européen Georges-Pompidou, 75015 Paris, France; 5 Department of Medical Oncology, University of Antwerp/Antwerp University Hospital, 2610 Wilrijk, Belgium; 6 Faculty of Science, RECETOX, Masaryk University, 625 00 Brno, Czech Republic; 7 Northern Ireland Cancer Centre, Belfast Health and Social Care Trust, Belfast, BT9 7 AB UK; 8 Patrick G. Johnston Centre for Cancer Research, School of Medicine, Dentistry and Biomedical Science, Queen’s University Belfast, Belfast, BT9 7AE UK; 9 Oncology Clinical Trials Office (OCTO), Department of Oncology, University of Oxford, Oxford, OX3 7LJ UK; 10 Centre for Statistics in Medicine, Nuffield Department of Orthopaedics, Rheumatology and Musculoskeletal Sciences, University of Oxford, Oxford, UK; 11 Department of Oncology &, University of Torino, Candiolo Cancer Institute, 10060 Candiolo, TO Italy; 12 Genomics, Diagnostics and Genomics Group, Agilent Technologies, 1831 Diegem, Belgium; 13 Institut National de La Sante Et de La Recherche Medicale (INSERM), Universite Paris Descartes, 75006 Paris, France; 14 Department of Molecular and Clinical Cancer Medicine, University of Liverpool, Ashton St, Liverpool, L69 3GE UK; 15 Cardiff University and Velindre University NHS Trust, Cardiff, CF14 2 TL UK; 16 Royal College of Surgeons in Ireland University of Medicine and Health Sciences, 123 St. Stephen’s, Green, Dublin, Ireland; 17 Department of Oncology, Molecular Biotechnology Center, University of Torino, Turin, Italy; 18 IFOM ETS, the AIRC Institute of Molecular Oncology, Milan, Italy

**Keywords:** RAS mutant, Colorectal cancer, Phase I, Binimetinib, Crizotinib, Pharmacokinetics, Pharmacodynamics, MET biomarker, CtDNA

## Abstract

**Background:**

Targeting *RAS* mutant (MT) colorectal cancer (CRC) remains a difficult challenge, mainly due to the pervasiveness of RAS/MEK-mediated feedback loops. Preclinical studies identified MET/STAT3 as an important mediator of resistance to KRAS-MEK1/2 blockade in *RAS*MT CRC. This dose escalation/expansion study assessed safety and initial efficacy of the MEK1/2 inhibitor binimetinib with MET inhibitor crizotinib in *RAS*MT advanced CRC patients.

**Methods:**

In the dose escalation phase, patients with advanced solid tumours received binimetinib with crizotinib, using a rolling- 6 design to determine the maximum tolerable dose (MTD) and safety/tolerability. A subsequent dose expansion in *RAS*MT CRC patients assessed treatment response. Blood samples for pharmacokinetics, MET biomarker and ctDNA analyses, and skin/tumour biopsies for pharmacodynamics, c-MET immunohistochemistry (IHC), *MET *in situ hybridisation (ISH) and *MET* DNA-ISH analyses were collected.

**Results:**

Twenty patients were recruited in 3 cohorts in the dose escalation. The MTD was binimetinib 30 mg B.D, days 1–21 every 28 days, with crizotinib 250 mg O.D continuously. Dose-limiting toxicities included grade ≥ 3 transaminitis, creatinine phosphokinase increases and fatigue. Thirty-six *RAS*MT metastatic CRC patients were enrolled in the dose expansion. Pharmacokinetic and pharmacodynamic parameters showed evidence of target engagement.

Across the entire study, the most frequent treatment-related adverse events (TR-AE) were rash (80.4%), fatigue (53.4%) and diarrhoea (51.8%) with grade ≥ 3 TR-AE occurring in 44.6%. Best clinical response within the *RAS*MT CRC cohort was stable disease in seven patients (24%). Tumour MET super-expression (IHC H-score > 180 and *MET* ISH + 3) was observed in 7 patients (24.1%), with *MET*-amplification only present in 1 of these patients. This patient discontinued treatment early during cycle 1 due to toxicity. Patients with high baseline *RAS*MT allele frequency had a significant shorter median overall survival compared with that seen for patients with low baseline *KRAS*MT allele frequency.

**Conclusions:**

Combination binimetinib/crizotinib showed a poor tolerability with no objective responses observed in *RAS*MT advanced CRC patients.

EudraCT-Number: 2014–000463 - 40 (20/06/2014: A Sequential Phase I study of MEK1/2 inhibitors PD- 0325901 or Binimetinib combined with cMET inhibitor Crizotinib in RAS Mutant and RAS Wild Type with aberrant c-MET).

**Supplementary Information:**

The online version contains supplementary material available at 10.1186/s12885-025-14068-1.

## Introduction

Patients with *RAS* mutant (MT) advanced colorectal cancer (CRC) exhibit poorer clinical outcomes, compared to their wild type (WT) counterparts, particularly in the metastatic setting [1]. Aberrant RAS pathway activation interrupts upstream receptor-tyrosine kinase (RTK) signalling, resulting in resistance to anti-EGFR therapies [2]. Outside a clinical trial, current therapy options for *RAS*MT CRC are primarily based on combinations of 5-FU with irinotecan (FOLFIRI) or oxaliplatin (FOLFOX) with/without anti-angiogenic agents [3]. Despite these treatments, median overall survival (OS) for *RAS*MT advanced CRC patients remain around 16–23 months and further systemic options are limited upon progression [1, 4, 5].

Mutant GTP-bound *RAS* can drive aberrant downstream signalling, mediated by an array of effectors. Raf/MEK/ERK signalling is considered to be a major RAS effector pathway, but single-agent activity for MAPK inhibitors in *KRAS*MT CRC has shown to be ineffective [6, 7]. Acute activation of pro-survival pathways and other adaptive resistance mechanisms [8] may limit success of single agent MAPK inhibition in the clinic. Given the known RAS signalling crosstalks and adaptive feedback loops [9], horizontal dual inhibition of MEK and PI3 K pathways has been trialled extensively with no anti-tumour activity observed, in part due to poor pharmacodynamic (PD) effects and high toxicity [10–14]. Several research groups, including our own, have shown a role for MET/STAT3 in regulating sensitivity to MEK1/2 inhibition in *RAS*MT/*BRAF*MT preclinical models [15–20]. c-MET pathway activation plays an essential role in the development/progression and drug-resistance of many cancers [21], and can be caused by MET protein and/or gene overexpression, gene amplification, MET exon- 14 skipping mutations and/or aberrant paracrine/autocrine HGF production [22].

Binimetinib (formerly MEK162) is a highly potent, selective, non-ATP-competitive oral small molecule inhibitor (SMI) of MEK1 and MEK2 [23, 24]. The target recommended phase 2 dose (RP2D) of binimetinib for combination studies with targeted agents is 45 mg B.D. Binimetinib in combination with the BRAF inhibitor encorafenib has been approved by the Food and Drug Administration for the use in *BRAF*V600EMT melanoma and non-small-cell lung cancer (NSCLC) [25, 26]. Crizotinib (formerly PF- 02341066), is an oral ATP-competitive SMI of c-MET, ALK and ROS1 [27], with a recommended dose of 250 mg B.D for patients with ROS1 + or ALK + metastatic NSCLC [28]. Our initial phase Ia study in patients with advanced solid cancers showed that combined MEK1/2 inhibitor PD- 0325901 with c-MET inhibitor crizotinib was safe; the maximum-tolerated dose (MTD) of crizotinib with PD- 0325901 was 200 mg B.D [29]. The development of PD- 0325901 was discontinued by the manufacturer, so an alternative MEK inhibitor was needed to continue the clinical investigation of MEK/MET combination.

On the basis of our preclinical data [20], the clinical significance of both c-MET and MEK1/2 pathways and the initial safety study with combined PD- 0325901/crizotinib [29], we selected binimetinib, a MEK1/2 inhibitor in late phase II/III development (at the time of trial development) and crizotinib for further clinical investigation. The MEK and MET Inhibition in Colorectal Cancer (MErCuRIC) study aimed to investigate the MTD, RP2D and safety/tolerability during the dose escalation, and evaluate preliminary anti-tumour activity of combined binimetinib/crizotinib treatment in *RAS*MT CRC patients in the phase Ib study. Secondary objectives were evaluation of pharmacokinetics (PK) and pharmacodynamics (PD). Exploratory endpoints included MET tumour, ctDNA and RNA sequencing analyses and initial correlation with treatment response.

## Methods

### Study design and treatments

The MErCuRIC study was an open-label, single arm phase I trial, conducted in 8 European centres (ClinicalTrials.gov number: NCT02510001).

Initial phase I monotherapy studies with binimetinib have shown that doses of binimetinib ≥ 30 mg B.D achieved plasma concentrations required to inhibit pERK1/2, and 45 mg B.D was identified as the RP2D in these studies [30, 31]. Hence, doses of 30–45 mg B.D of binimetinib were planned to be evaluated in the dose escalation study (Supplementary Fig. S1A). Based on the data from our initial dose escalation study with PD- 0325901 and crizotinib [29], dose level 5 started at crizotinib 200 mg B.D (Supplementary Figs.1 A-B). The recommended oral dose for crizotinib is 250 mg B.D [32], and was included to be investigated (dose level 7), if toxicity profile would permit.

A rolling-six design was employed [33]. The study design consisted of 3 pre-defined dose levels (Supplementary Fig. S1A). Dose levels 5a and 6a were included to enable exploration at O.D dosing if B.D dosing of crizotinib in combination was not well tolerated. There was also consideration given to the requirement to reduce the frequency of binimetinib dosing schedule from continuous dosing to days 1 to 21 every 28 days (Supplementary Fig. S1C). Intermittent dosing of binimetinib (days 1 through 21 of a 28-day cycle as opposed to a continuous schedule) has previously been trialled to enable patients to manage dosing [34]. The primary objective of the dose escalation Ia was to determine the MTD of crizotinib/binimetinib and to evaluate safety profile and dose limiting toxicities (DLT). Secondary endpoints included to define the recommended phase 1b dose (RPII), evaluate pharmacokinetics (PK), pharmacodymamics (PD) and anti-tumour activity. The MTD was defined as the highest dose of crizotinib and binimetinib at which no more than one of six patients experienced a DLT, which includes the assessment of safety and toxicity in cycle 1 (C1). Patients could remain on combination treatment until disease progression or predefined unacceptable toxicity. DLTs were defined as almost certainly or probable treatment-related adverse events (TR-AE) to either drug (Supplementary Table S1), during the first cycle of treatment.

The MTD was used as the recommended phase 1b (RPII) dose for the expansion phase of the study. This phase of the study was organised over 2 phases with patients only recruited to stage 2 if evidence of responsiveness was shown at stage 1 (Supplementary Fig. S1D). The primary objective of the dose expansion Ib was to assess treatment response (RECIST v1.1). Secondary objectives included progression-free-survival (PFS) and OS, characterization of TR-AEs, PK and treatment-mediated changes in MAPK and MET pathways (pERK; pSTAT3). Exploratory objectives were to assess tumour MET expression levels, ctDNA levels and transcriptional profiling and correlate with response to therapy.

### Patient selection

For the dose escalation, eligible patients were ≥ 16 years old, had advanced solid tumours, Eastern Cooperative Oncology Group (ECOG) performance status (PS) of 0–1, a life expectancy of > 3 months and adequate organ function. Key exclusion criteria included a history of hypoalbuminaemia, the presence of ascites/pleural effusions requiring taps, untreated or unstable brain metastases, a past history of retinal vein occlusion, intraocular pressure > 21 mmHg or increased risk of retinal vein thrombosis. Patients were also excluded if they had received previous treatment with HGF/c-MET or MEK1/2 inhibitors. For the dose expansion, eligible participants had histologically-confirmed advanced *RAS*MT CRC, were willing to undergo a biopsy for assessment of c-MET status and had at least one measurable lesion (RECIST v1.1).

### Safety and efficacy assessments

Safety assessments, ophthalmic/cardiac examinations were performed as previously described [29]. AEs were graded according to the National Cancer Institute Common Terminology Criteria for AE, version 4.03. Anti-tumour activity was conducted at baseline (within 28 days prior to C1/day 1 (D1)) and then every 2 cycles and evaluated by RECIST v1.1.

### Pharmacokinetics

The concentrations of crizotinib, binimetinib and its primary metabolite AR00426032 in plasma were measured in 18 and 26 patients in dose escalation and expansion phases respectively. Plasma samples were collected pre-dose and 1, 2, 4, 6, 8, 10 and 24 h after the dose on C1/D21. PK trough samples (pre-dose; 2 h post-dose) were obtained on day 21 of cycles 2, 4, 6, 8, 10 and 12. PK analyses were performed to ensure that the putative target levels of each drug to inhibit p–c-MET and pERK1/2 levels were reached with the combination treatment. Plasma concentrations of binimetinib/AR00426032 and crizotinib were determined using a validated high performance liquid chromatography mass spectrometry (HPLC–MS/MS) and carried out by QPS (Newark, USA) and Covance Laboratories (Indianapolis, USA) respectively.

### Pharmacodynamics

All patients in the dose escalation phase consented to a fresh frozen punch skin biopsy during screening and on C1/D15 (± 7 days). Fresh frozen skin and tumour biopsies during screening and on C1/D15 (± 7 days) were also required for the first 13 patients in the dose expansion phase. PD markers of MEK1/2 inhibition (pMEK1/2; pERK1/2) in skin biopsies and MET inhibition (pSTAT3) in tumour biopsy were assessed by Western blotting (WB), as previously described [20, 35]. Densitometry on WB images was performed using ImageJ software.

### Biomarker analysis

*RAS* mutational status (KRAS codons 12/13/61/117/146; NRAS codon 12/13/61/117/146) was determined on the archival tumour tissue by local testing.

MET expression was assessed on the pre-treatment metastasis biopsies or archival tumour tissues for the patients in the dose expansion cohort, using c-MET immunohistochemistry (IHC), *MET *in situ hybridisation (ISH) and *MET* DNA-ISH assays, as previously described [36]. A c‐MET IHC protein H-Score was obtained based on staining intensity (from 0 to 3) and staining extent (maximum 100%). MET super-expressor was defined as IHC H-score > 180 and *MET* ISH + 3 (Supplementary Fig. S2). Plasma samples (pre-dose; 6 h post-dose) to detect soluble MET levels were obtained on Cycles 1–6/Day 1 and Cycles 1–2/Day 15. Soluble MET levels were measured using a commercially available enzyme-linked immunosorbent assays (ELISA, Invitrogen) and carried out by QPS (Newark, USA).

ctDNA analysis was performed on plasma samples collected from patients in the dose expansion cohort. Plasma samples (pre-dose) for extraction of ctDNA were obtained on D1 of each cycle. ctDNA was extracted using the QIAamp MinElute ccfDNA Kit (Qiagen, Milan, Italy) according to the manufacturer’s instructions. Mutational analysis of *KRAS* and *NRAS* was performed by droplet digital PCR (ddPCR), according to manufacturer’s protocol using ddPCR Supermix for Probes (Bio-Rad, Segrate, Italy) and *KRAS* and *NRAS* assays. The results were reported as the percentage or fractional abundance of mutant DNA alleles to total (mutant plus wild-type) DNA allele, as previously described [37]. NGS analysis was performed using a liquid biopsy target panel, designed on hotspot regions of 44 genes relevant for CRC (Supplementary Fig. S3 A) and the Illumina NextSeq 500 sequencer with High Output 300 cycles v2 Kit (Illumina, CA, United States).

RNA and DNA extraction from fresh frozen tumour biopsies obtained at screening and C1D15, was performed using the Qiagen's DNA and RNA extraction kit (all prepDNA/RNA/miRNA universal kit). RNA sequencing was performed using the QuantSeq 3’ mRNA-Seq Library Prep Kit FWD for Illumina QuantSeq 3′ mRNA-Seq Library Prep Kit FWD (Lexogen, Vienna, Austria) and the Nextseq 500 sequencer. To predict CMS (consensus molecular subtypes) subtypes in the samples, we used the multi-class classifier « CMSclassifier» which is downloadable as an R package (https://github.com/Sage-Bionetworks/crcsc). The CRISclassifier R package was downloaded from Isella et al. [38] and implemented using the Nearest Template Prediction method.

### Statistical analysis

Safety and efficacy data were summarised using descriptive statistics. Evaluable patients for toxicity were those patients that received at least one dose of one or both drugs. Evaluable patients for MTD or dose escalation were those patients who completed C1 or withdrew early for experiencing a DLT. Response analyses (RECIST v1.1) were performed on an intention-to-treat basis, and any patient who received any dose of study treatment was evaluable for response. PFS was defined as the time between receiving the first dose of study medication to disease progression or death from any cause. OS was defined as the time between C1D1 to death from any cause. PFS and OS were estimated using the Kaplan–Meier method. Statistical significance was calculated from distinct technical replicates by Student’s t-test (2-tailed, 2 sample equal variance on unpaired data), in GraphPad Prism 8. Graphs were plotted as means with error bars represented as SD; statistical significance was denoted as follows: *** = *p* < 0.001, ** = *p* < 0.01, * = *p* < 0.05, ns = *p* > 0.05.

## Results

### Baseline demographics

A total of 20 eligible patients with advanced solid tumours were included in the dose escalation phase Ia. Patients’ characteristics are summarized in Table 1. CRC was the most common (60%) solid tumour type. Thirty-six patients with advanced *RAS*MT CRC were enrolled in the dose expansion phase Ib. Demographic and baseline characteristics are listed in Table 2. All phase Ia/Ib patients had an ECOG PS of 0 or 1. Patients were heavily pre-treated with 45% (dose escalation) and 58% (dose expansion) having received ≥ 4 prior anti-neoplastic regimens. In the dose expansion phase, 78% of patients had ≥ 4 organs involved with metastases and 64% of patients progressed in the first 3 months on their previous systemic treatment. The median baseline *RAS*MT allele frequency detected in plasma DNA was 24.93 GE/ml.

**Table 1:** Baseline patient demographic, characteristics and treatment allocation for patients in dose escalation phase Ia. Abbreviation: ECOG = Eastern Cooperative Oncology Group; PS = Performance status

**Cohort** binimetinib crizotinib	**Cohort 7** 30 mg B.D days 1–28 200 mg B.D (*n* = 8)	**Cohort 12** 30 mg B.D days 1–21 200 mg B.D (*n* = 5)	**Cohort 13** 30 mg B.D days 1–21 250 mg O.D (*n* = 7)	**Total** (*n* = 20)
**Demographic**				
**Age** (years)	51	55	60	55.3
median (range)	(33–72)	(40–65)	(46–70)	(33–72)
**Gender**				
Female, n (%)	3 (37.5)	3 (60)	0	6 (30)
Male, n (%)	5 (62.5)	2 (40)	7 (100)	14 (70)
**ECOG PS**				
0, n (%)	5 (62.5)	5 (100)	4 (57.1)	14 (70)
1, n (%)	3 (37.5)	0 (0)	3 (42.9)	6 (30)
**Median range of prior systemic therapies**				
0–1, n (%)	0	0	1 (14.3)	1 (5)
2–3, n (%)	4 (50)	3 (60)	3 (42.8)	10 (50)
4–5, n (%)	3 (37.5)	1 (20)	1 (14.3)	5 (25)
≥ 6, n (%)	1 (12.5)	1 (20)	2 (28.6)	4 (20)
**Tumour origin**				
Peritoneal mesothelioma, n (%)	1 (12.5)	0 (0)	0 (0)	1 (5)
Colorectal cancer, n (%)	5 (62.5)	3 (60)	4 (57.1)	12 (60)
Cervical cancer, n (%)	1 (12.5)	0 (0)	0 (0)	1 (5)
Cholangio Carcinoma, n (%)	1 (12.5)	0 (0)	0 (0)	1 (5)
Small Bowel cancer, n (%)	0 (0)	1 (20)	0 (0)	1 (5)
Pancreatic cancer, n (%)	0 (0)	1 (20)	2 (28.6)	3 (15)
Parotid adenocystic carninoma, n (%)	0 (0)	0 (0)	1 (14.3)	1 (5)

**Table 2:** Baseline demographic, characteristics for patients in the dose expansion Phase Ib study

**Demographics**	***RAS*MT CRC** (*n* = 36)
**Age** (years)	62
median (range)	(32–78)
**Gender**	
Male, n (%)	18 (50)
Female, n (%)	18 (50)
**ECOG**	
0, n (%)	17 (47.2)
1, n (%)	19 (52.8)
**Median range of prior systemic therapies**	
Range (Median)	1–10 (4.7)
0–1, n (%)	2 (5.6)
2–3, n (%)	13 (36.1)
4–5, n %	7 (19.4)
≥ 6, n (%)	14 (38.9)
**Recent systemic therapy duration (months)**	
Range (Median)	0.25–20 (3.7)
≤ 3 months, n (%)	23 (64)
4–6 months, n (%)	10 (28)
≥ 7 months, n %	3 (8)
**Tumour side**	
Right sided	5 (13.9)
Left sided	15 (41.7)
Unknown	16 (44.4)
**No. of organs involved with metastases** ≥ 2, n (%)	36 (100)
**No. of organs involved with metastases** ≥ 4, n (%)	28 (78)
Metastatic site locations	
Liver, n (%)	28 (78)
Lung, n (%)	26 (72)
Lymph node, n (%)	14 (39)
Peritoneum, n (%)	8 (22)
Bone, n (%)	5 (14)
Other, n (%)	8 (22)
**Time from initial diagnosis (months)**	
Range	12–110
Median	43.7
***RAS* mutational status**	
KRAS NS, n (%)	1 (2.8)
KRAS exon 2, n (%)	31 (86)
G12D	9 (25)
G12 V	6 (16.7)
G12 C	2 (5.6)
G12 A	2 (5.6)
G12S	1 (2.8)
G13D	10 (27.8)
NS	1 (2.8)
KRAS exon 4, n (%)	2 (5.6)
K117 N	1 (2.8)
A146 T	1 (2.8)
NRAS exon 3, n (%)	1 (2.8)
NRAS NS, n (%)	1 (2.8)
**Mean DNA (GE/ml)** ***RAS*** **mutation** **frequency baseline** Range Median	Range 1.6-86.3 Median 24.93

Abbreviation: *ECOG *Eastern Cooperative Oncology Group, *PS *Performance status, *NS *Not specified. *RAS* mutational status determined on archival tumour/pre-treatment biopsy

### Dose escalation

Three patients withdrew early from the dose escalation not for DLT, therefore 17/20 recruited patients were evaluable for MTD. Two DLT’s (grade 3 increased ALT/AST; grade 3 increased CPK) were observed in cohort 7, two DLT’s in cohort 12 (grade 3 increased ALT and grade 3 increased CPK) and 1 DLT in cohort 13 (grade 3 fatigue) (Supplementary Table S2). Dose level 5a* (binimetinib 30 mg B.D days 1–21 and crizotinib 250 mg O.D days 1–28) was therefore defined as the MTD and the recommended dose for further evaluation in the phase Ib component of the trial (Supplementary Fig. S1B-C).

### Treatment exposure

A total of 61 and 75 cycles of treatment were given in the phase Ia and phase Ib respectively, with a median of 3 cycles (range, 1–14) and 2 cycles (range, 1–6) per patient for phase Ia and phase Ib respectively. The most common reason for discontinuation of study treatment was disease progression (65% and 67% for phase Ia and phase Ib), while remaining reasons were toxicity (10% and 22% for phase Ia and phase Ib), investigator’s (10% for phase Ia) and patient’s decision (15% and 8% for phase Ia and phase Ib) and disease-related adverse events (3% for phase Ib).

### Safety

There were in total 210 drug-related adverse events (DR-AE) in the dose escalation phase, of which 200 were determined to be related to binimetinib and 164 related to crizotinib. Common DR-AEs, observed in ≥ 2 patients, are summarised in Table 3. The most common DR-AE’s were rash (95%), followed by fatigue (70%), diarrhoea (65%), nausea (45%), oedema (40%), CPK increases (40%), AST/ALT increases (25%), vomiting (20%), arthralgia/myalgia (20%), blurred vision (20%) and peripheral neuropathy (20%) (Table 3). Fifty-seven DR-AEs were observed in the 8 patients treated at the highest dose level (Supplementary Table S3), with rash being the most common DR-AE (100%). Most DR-AEs in all cohorts were of grade 1 or 2 and there were no deaths due to DR-AEs. The most common grade ≥ 3 biochemical DR-AEs were CPK and ALT/AST increases and grade ≥ 3 non haematological/biochemical DR-AEs were fatigue, oedema, decrease in left ventricular ejection fraction, pleural effusion, dyspnoea, postural hypotension and pneumonia, observed in 10 patients (50%) (Supplementary Table S3). A total of 14 serious AE (SAE) were reported in 11 patients. However, only four of these were grade 3 (lung infection, pneumonitis, dyspnoea, postural hypotension) and thought to be drug-related (Supplementary Table S2).

**Table 3:** Summary of treatment-related, non-hematologic and non-biochemical, biochemical and haematological adverse events (AE) occurring in ≥ 2 patients in dose escalation (All cohorts) and dose expansion phases who started treatment, by CTCAE grade

No. of patients AE affected	Dose escalation (*n* = 20)		Dose expansion (*n* = 36)	
	**Any grade n, (%)**	**Grade ≥ 3 n, (%)**	**Any grade n, (%)**	**Grade ≥ 3 n, (%)**
**Non-haematological and non-biochemical**				
Rash	19 (95)	0 (0)	26 (72.2)	1 (2.77)
Pruritus	0 (0)	0 (0)	5 (13.9)	0 (0)
Dry skin	0 (0)	0 (0)	3 (8.3)	0 (0)
Nausea	9 (45)	0 (0)	13 (36.1)	0 (0)
Vomiting	4 (20)	0 (0)	13 (36.1)	2 (5.55)
Dyspepsia	0 (0)	0 (0)	2 (5.55)	0 (0)
Dysgeusia	0 (0)	0 (0)	2 (5.55)	0 (0)
Dry Mouth	2 (10)	0 (0)	0 (0)	0 (0)
Mucositis (mouth)	2 (10)	0 (0)	0 (0)	0 (0)
Diarrhoea	13 (65)	0 (0)	16 (44.4)	0 (0)
Constipation	0 (0)	0 (0)	5 (13.9)	0 (0)
Abdominal pain	0 (0)	0 (0)	2 (5.55)	0 (0)
Oedema	8 (40)	1 (5)	7 (19.4)	1 (2.77)
Arthralgia/Myalgia	4 (20)	0 (0)	4 (11.1)	0 (0)
Anorexia	3 (15)	0 (0)	3 (8.3)	0 (0)
LV Ejection fraction ↓	2 (10)	1 (5)	4 (11.1)	1 (2.77)
Fatigue	14 (70)	1 (5)	16 (44.4)	1 (2.77)
Dyspnoea	2 (10)	1 (5)	3 (8.3)	1 (2.77)
Cough	2 (10)	0 (0)	0 (0)	0 (0)
Fever	0 (0)	0 (0)	2 (5.55)	1 (2.77)
Eye disorder (Blepharitis)	2 (10)	0 (0)	0 (0)	0 (0)
Blurred vision	4 (20)	0 (0)	3 (8.3)	1 (2.77)
Retinopathy/Retinal haemorrhage	0 (0)	0 (0)	4 (11.1)	1 (2.77)
Dizziness	2 (0)	0 (0)	0 (0)	0 (0)
Peripheral neuropathy	4 (20)	0 (0)	2 (5.55)	0 (0)
**Haematological and biochemical**				
Anaemia	0 (0)	0 (0)	5 (13.9)	1 (2.77)
Thrombocytopenia	0 (0)	0 (0)	2 (5.55)	0 (0)
CPK increase	8 (40)	3 (15)	9 (25)	2 (5.55)
ALT and/or AST increase	5 (25)	2 (10)	10 (27.8)	6 (16.7)
ALP increase	0 (0)	0 (0)	5 (13.9)	0 (0)
Hypoalbuminaemia	2 (10)	0 (0)	2 (5.55)	0 (0)
GGT increase	0 (0)	0 (0)	2 (5.55)	0 (0)

In the dose expansion phase, 297 DR-AE were reported, of which 288 were determined to be related to binimetinib and 244 related to crizotinib. The most common DR-AE were rash (72.2%), diarrhoea (44.4%), fatigue (44.4%), vomiting (36.1%), nausea (36.1%), ALT/AST increases (27.8%), CPK increases (25%) and edema (19.4%) (Table 3). The most common grade ≥ 3 DR-AE were ALT/AST and CPK increases, vomiting, rash, oedema, decreased LVEF, fatigue, dyspnoea, fever, blurred vision/retinopathy, photophobia and anaemia occurring in 15 (41.7%) patients. A total of 23 SAE were reported in 14 patients, 8 of these were grade ≥ 3 and thought to be drug-related (Supplementary Table S2). The most common drug-related SAE was AST/ALT increases.

### Pharmacokinetics

**Fig. 1:**
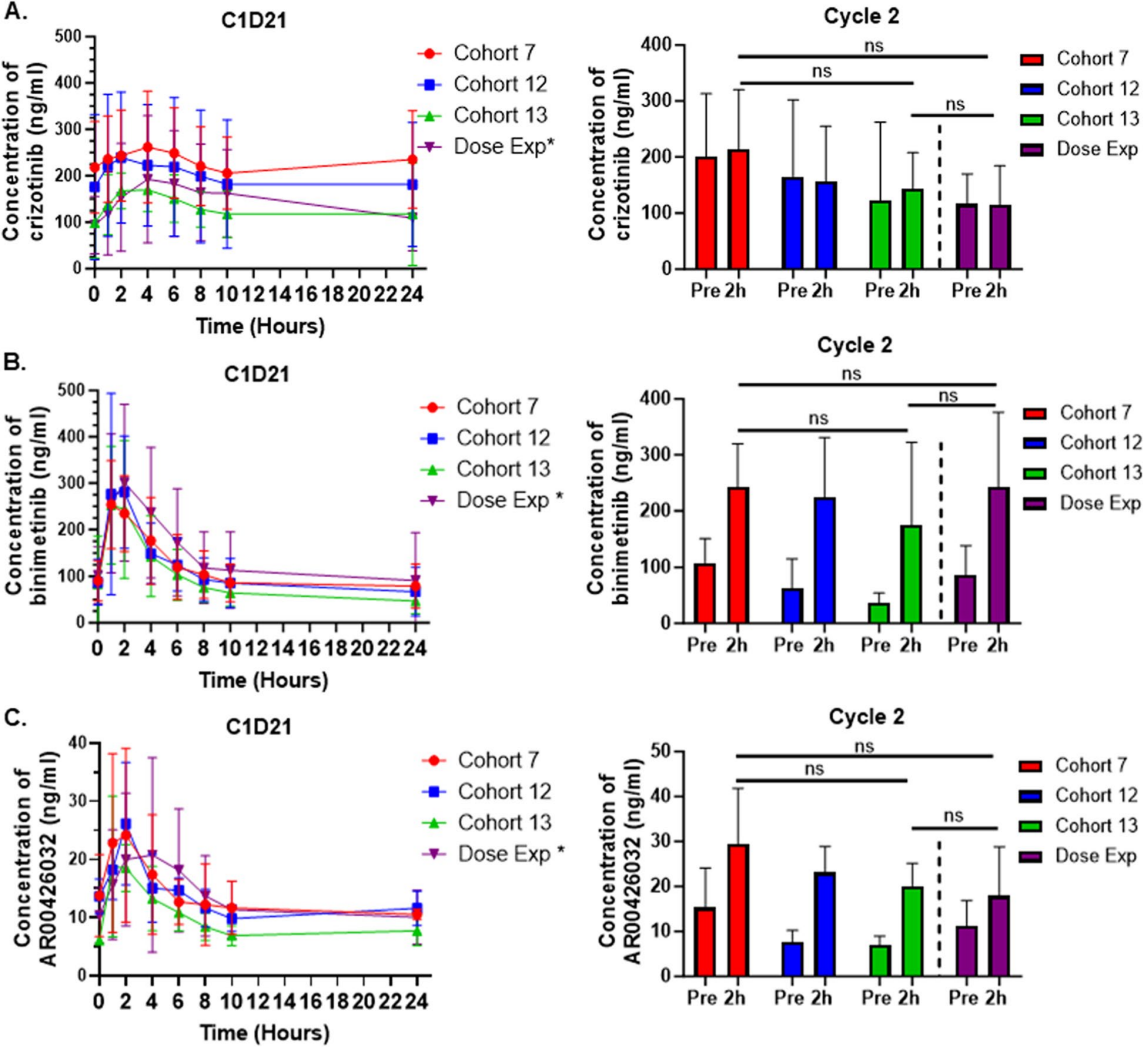
Plasma concentrations for crizotinib, binimetinib and AR00426032 for cohort 7, 12 and 13 dose escalation and dose expansion phases. Left: Twenty-four hours PK profiles for crizotinib, obtained at C1D21 (**A**), for binimetinib obtained at C1D21 (**B**) and for AR00426032 (**C**) obtained at C1D21. Star (left) and dotted line (right) indicates that PK samples for dose expansion were analysed at a separate time

The effects of binimetinib and crizotinib on PK parameters of crizotinib and binimetinib respectively were assessed in 18 patients at the 3 dose levels in the dose escalation phase (Fig. 1; Supplementary Table S4). Crizotinib was absorbed with peak plasma concentrations occurring within 0.97 h and 6.00 h after dosing, without significant differences between the 3 cohorts (*p* = 0.189). Twice-daily dosing of crizotinib in cohorts 7 and 12 resulted in mean C_min_ of 195 ± 101 ng/ml on C1D21 and pre-dose trough levels of 187 ± 116 ng/ml on C2D21, both values which are comparable to those published by Tan et al*.* [39] for crizotinib monotherapy. Twice-daily dosing of crizotinib did result in higher increases in C_max_, C_min_ and AUC_0–10 h_ in cohorts 7 and 12 on day 21 compared with cohort 13, although these differences were not statistically significant. Similar data were observed for the 2 h post-dose crizotinib concentrations on C2D21 (Fig. 1). For crizotinib, the PK parameters were comparable for cohorts 7 and 12 and were similar to those reported for cohorts 2–4 in our initial study [29]. For cohort 13, the PK parameters for crizotinib were similar to those reported for cohort 1 on C1D21 (2 mg PD- 0325901 B.D and 250 mg crizotinib O.D) [29].

After oral administration, binimetinib was absorbed rapidly, with peak plasma concentrations occurring within 0.98 h and 6 h after dosing. There was no significant difference in average time to reach C_max_ between cohort 7–12 and cohort 13 (Supplementary Table S4). There were also no significant differences in AUC_0–10 h_, C_max_, C_min_ or 2 h post-dose binimetinib concentrations between the different cohorts on C1D21 and C2D21 respectively. Median T_max_ and C_max_ were comparable to those observed in a previous study of single agent binimetinib for the same dose [31]. Similar data were obtained for AR0042603, a metabolite of binimetinib (Fig. 1; Supplementary Table S4). Taken together, these data suggests that co-administration of binimetinib with crizotinib has not affected the PK of binimetinib and crizotinib compared to single agent dosing of each agent.

In the dose expansion phase, plasma sample analysis was performed in 26 patients. For crizotinib, average values for the PK parameters were very similar to those measured for cohort 1 (2 mg PD- 0325901 B.D and 250 mg crizotinib O.D) [29] and cohort 13 (Fig. 1; Supplementary Table S4), which had the same dosing regimen for crizotinib. No remarkable differences in average PK parameters for binimetinib and AR00426032 were observed between dose expansion and three dose escalation cohorts. In addition, for binimetinib the values of T_max_ and C_max_ on C1D21 were similar to those previously reported by Bendell *et. al* for administration of binimetinib at 30 mg B.D on C1D15 [31]. These data supply more evidence that co-administration of binimetinib with crizotinib has not affected the PK of binimetinib compared to single agent dosing.

### Pharmacodynamics

**Fig. 2:**
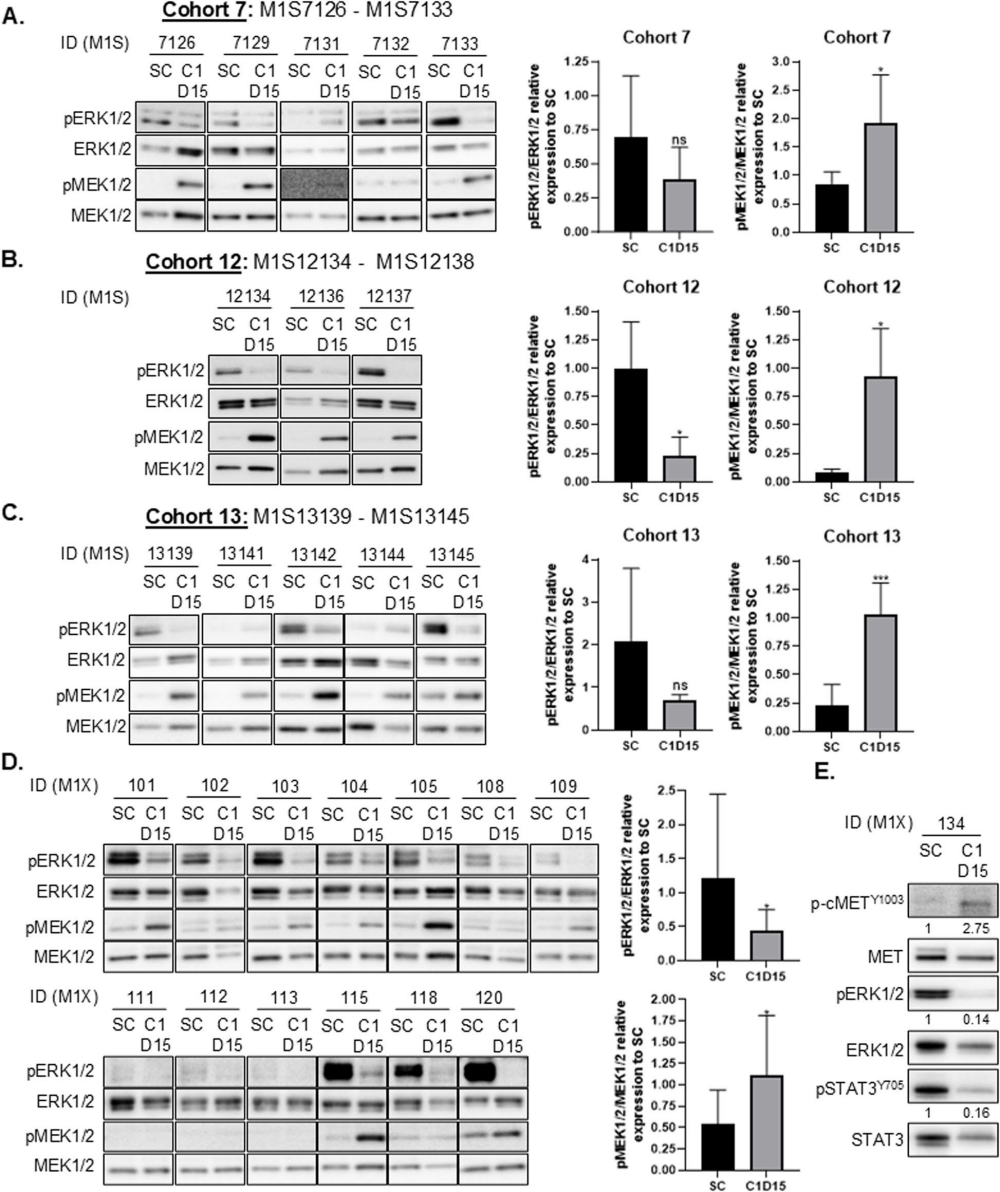
Modulation of pERK1/2^T202/Y204^, pMEK1/2^S217/221^, pMET^Y1003^ and pSTAT3^Y705^ expression levels in paired skin and/or tumour biopsies. **A-C**. Left: pERK1/2-ERK, pMEK1/2-MEK1/2 levels in paired skin biopsies dose expansion Cohort 7: binimetinib 30 mg B.D 1 - 28 d and crizotinib 200 mg B.D continuously (**A**), Cohort 12: binimetinib 30 mg B.D 1 - 21 d and crizotinib 200 mg B.D continuously (**B**), Cohort 13: binimetinib 30 mg BD 1 - 21 d and crizotinib 250 mg O.D continuously (**C**). **A-C** Right: Densitometry was performed on the WB images using ImageJ software. SC = screening. C1D15: Skin biopsy obtained between 3–6 h following morning dose of binimetinib. **D**. Left: pERK1/2^T202/Y204^ and pMEK1/2^S217/221^ levels in paired skin biopsies dose expansion. Right: Densitometry was performed on the WB images shown in A using ImageJ software. SC = screening. C1D15: Skin biopsy obtained between 3–6 h following morning dose of binimetinib. **E** pcMET^Y1003^, pSTAT3^Y705^, pERK1/2^T202/Y204^ expression and phosphorylation in paired tumour biopsies. Densitometry was performed on the WB images for pcMET^Y1003^, pSTAT3^Y705^, and pERK1/2^T202/Y204^ using ImageJ software. SC = screening. C1D15: Tumour biopsy obtained between 3–6 h following morning dose of binimetinib and crizotinib

Expression of phospho-ERK1/2^T202/Y204^ and phospho-MEK1/2^S217/221^ was evaluated in pre-treatment and post-treatment skin biopsies from 13 patients in the dose escalation phase (Fig. 2 A-C). Binimetinib treatment resulted in a significant accumulation of catalytically-inactive pMEK1/2 [40], in cohort 7, 12 and 13 patients. Densitometry analyses also showed a marked reduction in pERK1/2 levels following 15 days of combined binimetinib/crizotinib treatment in 77% of patients, but this was only significant in cohort 12.

Phospho-ERK1/2^T202/Y204^ and phospho-MEK1/2^S217/221^ levels were also significantly decreased and increased respectively in all post-treatment skin biopsies collected in the dose expansion phase (Fig. 2D). In tumour samples from one patient from whom a paired biopsy was available, pERK1/2 levels were also markedly decreased (Fig. 2E). In addition, increased pMET^Y1003^ and decreases in pSTAT3^Y705^ levels were also observed during treatment, providing evidence of strong target engagement and pathway suppression for crizotinib (Fig. 2E) [22].

### Efficacy

**Fig. 3:**
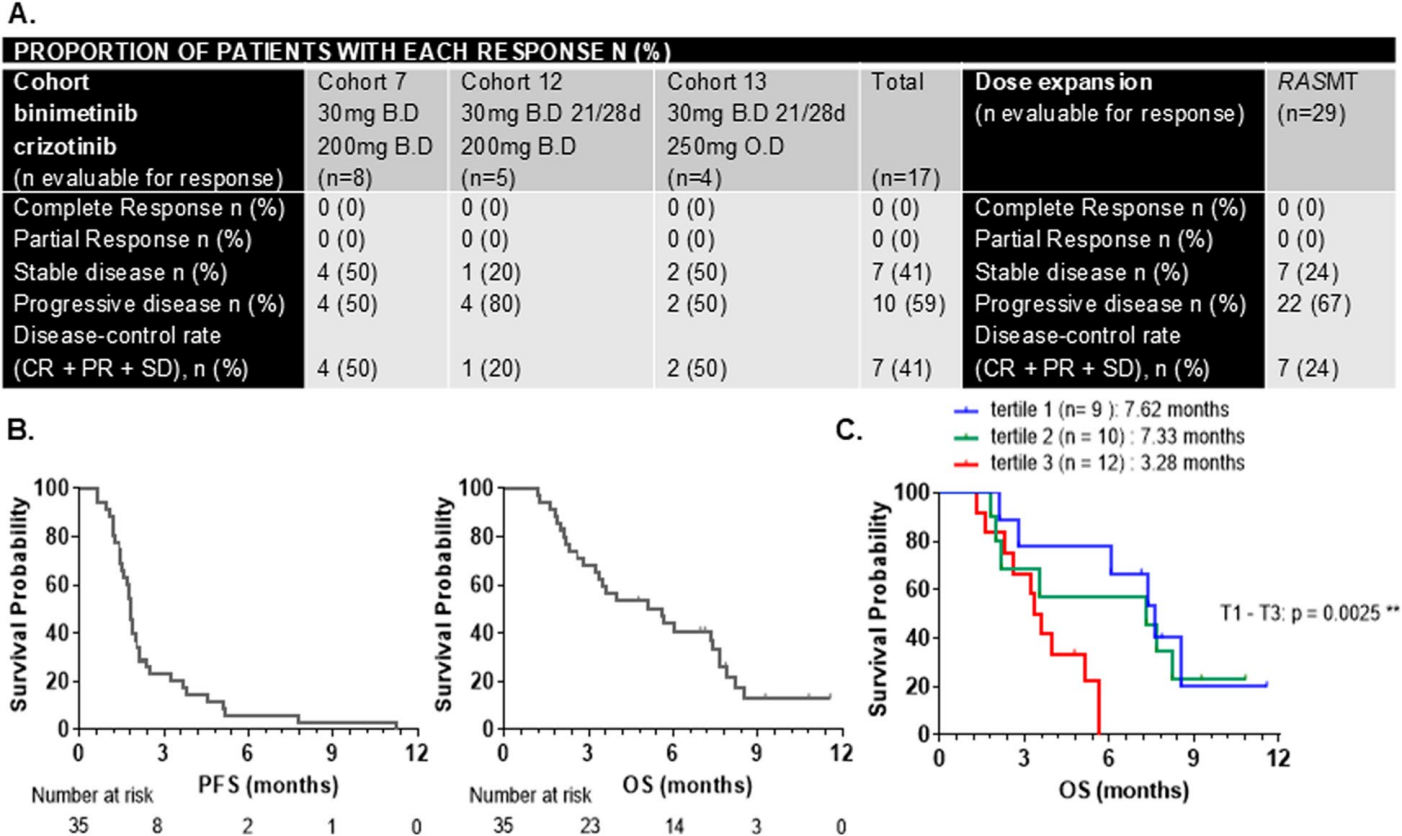
Tumour response, progression-free survival, and overall survival for combined binimetinib with crizotinib. **A**. Best radiological response observed to treatment as per cohort in dose escalation phase and phase Ib *RAS*MT cohort. **B**. Kaplan–Meier curves for median progression-free survival (PFS) and median overall survival (OS) in the phase Ib study. Left: Median PFS for all patients is 1.81 months (95% CI 1.51–2.04 months). The survivor function is 0.06 at 6 months (95% CI 0.01–0.17). Right: Median OS for all patients is 5.62 months (95% CI 2.97–7.40 months). The survivor function is 0.44 at 6 months (95% CI 0.27–0.60). **C.** Kaplan–Meier curves for median overall survival in patients from the dose expansion phase with low (tertile 1), median (tertile 2) and high (tertile 3) baseline *RAS* mutant allele frequency in circulating tDNA. 1 st tertile: 1.6–10.5; 2nd tertile: 13.57–32.15 and 3rd tertile: 34–17–86.33

Of 17 evaluable patients in the dose escalation phase, 7 patients (41%) had radiologically stable disease (Fig. 3A) and one patient had a prolonged disease stabilization for 15 cycles. In the expansion phase, 29 of the 36 patients were evaluable for response. No objective responses were observed; however, 7 patients (24%) had a disease stabilization (Fig. 3A). Median PFS on treatment was 1.81 months, and the most common reason for discontinuing treatment was disease progression. Median OS was 5.62 months (Fig. 3B, left and right).

### MET Biomarker analysis

MET protein, RNA-ISH and DNA-ISH were performed on the pre-treatment metastasis biopsies or archival tumour tissues in the 36 patients (100%) recruited to the dose expansion cohort (Supplementary Fig. S2 A). Variable *MET* RNA‐ISH and c‐MET IHC protein levels were observed within the cohort. In contrast, DDISH analysis determined that *MET* amplification was present in only one patient (2.8%). *MET* RNA‐ISH expression demonstrated a moderate positive correlation with increasing c‐MET IHC protein expression (*P* < 0.0005). The patient with *MET* amplification was found to have *MET* RNA‐ISH + 3 and c‐MET IHC 280 scores (super-expressor). A statistically non-significant trend for higher c-MET IHC H-score was observed in the patients with progressive disease (Supplementary Fig. S2B).

To identify possible predictive markers for crizotinib treatment, analysis of soluble (decoy) c-MET levels was performed (Supplementary Fig. S2 C). Soluble c-MET levels were markedly increased pre-C2D1 and D15 compared to the levels measured at pre-C1D1. Furthermore, treatment with combined crizotinib/binimetinib did not affect the soluble c-MET levels. Consistent with previous data [41], no correlation between the soluble c-MET levels and tissue c-MET IHC levels in 36 patients in the dose expansion cohort was found. Furthermore, no significant difference in soluble c-MET levels between groups with stable disease and progressive disease was observed. Taken together, the predictive ability of plasma/tissue cMET levels for the efficacy of crizotinib treatment was not evident.

### CMS/CRIS groups and response to combined binimetinib/crizotinib

Screening biopsies from metastatic lesions from 19 patients (53%) in the dose expansion phase were available for CMS/CRIS sub-classification (Supplementary Fig. S4 A). The distribution of the different CMS groups was CMS1 15.8% (*n* = 3), CMS2 63.2% (*n* = 12) and CMS4 21% (*n* = 4). The distribution of CMS groups on C1D15 (± 7 days) (*n* = 14) was CMS2 78.5% (*n* = 11) and CMS4 21.5% (*n* = 3). Liver metastases were predominantly CMS2, lung metastases mainly CMS1 and the subcutaneous metastasis was CMS4 (Supplementary Fig. S4B). A lack of CMS3 subtype was seen in this *RAS*MT patient cohort, which is known to strongly associate with that subtype [42]. Furthermore, CMS1 tumours displayed upregulation of immune genes, associated with microsatellite instability, but was also defined by metabolic dysregulation. CMS2 displayed epithelial differentiation and strong upregulation of WNT, MYC downstream targets and cell cycle genes, whereas CMS4 showed clear upregulation of genes implicated in EMT and TGF-β signalling (Supplementary Fig. S4 C). There was a trend for higher MET protein/RNA-ISH in CMS4 tumours (Supplementary Fig. S3B).

The distribution of CRIS groups was CRIS-A 16% (*n* = 3), CRIS-B 21% (*n* = 4), CRIS-C 10.5% (*n* = 2), CRIS-D 26.25% (*n* = 5), CRIS-E 26.25% (*n* = 5) (Supplementary Fig. S4 A). The distribution of CRIS groups on C1D15 (± 7 days) was CRIS-C 35.7% (*n* = 5), CRIS-D 42.9% (*n* = 6), and CRIS-E 21.4% (*n* = 3). There was no clear correlation between CMS and CRIS classifications, as expected (Supplementary Fig. S4D). CMS classification in screening and C1D15 biopsies remained stable in 78% of cases, whereas CRIS classification changed between screening and C1D15 biopsies. Fisher exact tests showed no association between CMS or CRIS groups and response to treatment (*p* = 0.69 and *p* = 0.59, respectively).

#### Analysis of plasma *RASMT* allele frequency and association with overall survival

ctDNA analysis was performed on plasma samples collected from 34/36 (94%) patients from the dose expansion cohort. Mutational analyses of KRAS and NRAS were performed by ddPCR. Comparison of patient-matched fresh plasma and available tumour samples showed identical hotspot mutation in 29 (97%) of 30 patients for *KRAS* (Supplementary Table S5)*.* High allelic frequency of MT *KRAS* G12, G13D, A146 and *NRAS* Q61 were detected in plasma samples from 32 of 34 analysed patients. High baseline *RAS*MT allele frequency was also associated with a significant shorter median OS compared with that seen for low baseline *RAS*MT allele frequency in crizotinib/binimetinib-treated patients (3.28 m vs. 7.62 m; *p* = 0.0025) (Fig. 3C; Supplementary Fig. S3B).

In order to discover secondary resistance mutations occurring following crizotinib/binimetinib treatment, we selected plasma samples from patients that received at least 4 cycles and progressed afterwards (Supplementary Fig. 3 C)*.* In all 3 patients, we identified the same mutations in C1D1 and end-of-treatment samples, and we also confirmed the *KRAS* mutations.

## Discussion

While significant progress has been made in the treatment of specific genetic subgroups, such as *RAS/BRAF*WT [43], *BRAF*MT [44] and MSI-H CRC [45], an effective therapeutic strategy for *RAS*MT advanced CRC, the most common oncogenic driver in CRC (~ 45–50%) is still lacking. Recently, inhibitors [eg. sotorasib (AMG510) and adagrasib (MRTX849)] that covalently bind to the cysteine of the glycine- 12-cysteine (G12 C) substitution of *KRASG12*MT, reported in 2–4% of mCRC [46], in combination with EGFR monoclonal antibodies (CodeBreaK300 [47]; KRYSTAL- 1 [48] have shown promising results in heavily pre-treated CRC patients. Other approaches to target the more common KRAS mutations (eg. G12D—MRTX1133 [49]), pan-(K) RAS inhibitors (eg. BI- 2865, RMC- 6236 [50, 51]) and inhibitors for the RAS guanine nucleotide exchange factor SOS1 [52] are investigated at preclinical/first-in-human stages in pan-cancer models. Therapeutic targeting of the RAS downstream effector MEK1/2, has shown limited activity in *KRAS*MT advanced CRC [6]. This is the first phase 1a/b study evaluating the safety and efficacy of MEK1/2 inhibitor binimetinib with c-MET inhibitor crizotinib, and was supported by preclinical data showing synergy between MEK1/2 and MET inhibition in *KRAS*MT CRC models [20]. The activity we observed in *KRAS*MT CRC in vivo models appeared for a major part driven by MEK1/2 inhibition [20]. Therefore, based on these data, we attempted to prioritize maintenance of MEK1/2 blockade, while also attempting to combine and maintain MET inhibition. The trial highlights the many challenges of combining targeted agents [53]. The particular challenge of combining MEK1/2 and MET inhibition included known overlapping monotherapy toxicities. Indeed, asymptomatic grade 3 increases in AST/ALT and CPK levels resulted in the need to change schedule of administration of binimetinib (cohort 12) and de-escalate the dose of crizotinib (cohort 13) from recommended monotherapy doses [31, 32] to mitigate these tolerability issues. The phase Ia dose-escalation phase met its primary objective of establishing the MTD of crizotinib/binimetinib and to evaluate safety profile and dose limiting toxicities (DLT). MTD and schedule was defined to be 30 mg binimetinib B.D (days 1–21) and 250 mg crizotinib O.D continuously in a 28-day cycle.

Consistent with the known class effects of MEK1/2 [7] and MET inhibition [54] and data from our initial phase 1a trial with PD- 0325901 and crizotinib [29], the most commonly reported DR-AEs across all dose levels in the dose-finding phase 1a were rash (95%), fatigue (70%) and diarrhoea (65%). The most common ≥ grade 3 DR-AEs were asymptomatic increases in CK and ALT/AST and were considered to be mostly related to binimetinib [31]. The PK data of the dose escalation phase suggested no drug-drug interaction between binimetinib and crizotinib. Importantly, plasma concentrations of binimetinib reached levels consistent with those required to inhibit MEK1/2 activity and inhibited pERK1/2 levels in all 3 cohorts. Although not significant, once-daily dosing of crizotinib (cohort 13), resulted in lower plasma concentrations, C_min_ and AUC_0–10 h_ compared with twice-daily dosing (cohorts 7 and 12), values that were comparable to the previously reported data for cohort 1 with PD- 0325901 and crizotinib [29]. Nevertheless, the median trough plasma concentrations of crizotinib observed in cohort 13 was in excess of 62 ng/mL, the pre-clinically predicted effective concentration to inhibit cMET [39]. In terms of efficacy, the phase 1a part showed limited anti-tumour activity in these heterogeneous and biomarker-unselected participants, similar to our clinical trial with crizotinib and PD- 0325901 [29]. One patient with advanced parotid adenocystic carcinoma, whose tumour had a mutation in *HRAS* remained on treatment with stable disease for 15 cycles; this is interesting in the context that *HRAS* has been proposed as a target for MEK1/2 inhibitors [55].

The expansion phase of MErCuRIC investigated binimetinib and crizotinib in *RAS*MT CRC patients. Despite choosing the lower dose of crizotinib (250 mg O.D), grade ≥ 3 TR-AE were reported in 44.4% of patients, resulting in both dose interruptions and reductions, suggesting that the overall tolerability of combined binimetinib/crizotinib may be challenging. These classes of agents have some overlapping toxicities including fatigue, nausea/vomiting, diarrhoea, peripheral oedema, and liver function disturbances [31, 56], likely limiting the ability to dose both agents continuously. Noteworthy, other combination regimens using HGF/MET monoclonal antibodies in combination with other TKI’s (eg. Erlotinib) reported improved/acceptable tolerability [57].

Although dual MEK1/2 and MET pathway inhibition had strong preclinical rationale in *KRAS*MT CRC [20], the clinical efficacy was limited and does not support combined binimetinib/crizotinib in unselected, heavily pre-treated *RAS*MT advanced CRC patients. The *KRAS*MT MET superexpressor subgroup was very small (7 patients). Only 1 of these patients had a *MET*-amplified tumour, who discontinued treatment early during Cycle 1 due to grade 3 nausea. While it is possible that the limited dataset and tumour heterogeneity contributed to the lack of meaningful observed activity, it is also likely that the inability to combine these two agents at optimal doses underlies the lack of clinical activity. Furthermore, although all patients shared a common driver alteration in *RAS*, different coexisting mutations (eg. *TP53*) and CMS categories might explain the differences in responses between the CRC tumours [58]. It is possible that higher doses of crizotinib and binimetinib would provide better results in terms of depth of inhibition of the critical cellular pathways and clinical activity, but this could not be achieved due to the reported tolerability issues. Recently, the first-in-human study of ABBV- 400, a novel cMET–targeting antibody–drug conjugate, showed a manageable safety profile with enriched responses in a cMET-high mCRC subpopulation [59]. Therefore, it is also possible that these more novel MET monoclonal antibodies (mAbs) (eg. ABBV- 400, Telisotuzumab vedotin [57], TR1801-ADC [60]) might show improved tolerability when combined with MEK1/2 inhibitors, more optimal pathway modulation and improved clinical activity in MET-dependent *RAS*MT CRC patients. Another way of overcoming the toxicities that we experienced is to evaluate alternative schedules eg. pulsatile/intermittent dosing [53, 61]. This strategy was successfully explored in a study of the MEK1/2 inhibitor AZD6244 with the AKT inhibitor MK- 2206 [10], where pulsatile dosing of MK- 2206 seemed to be better tolerated in the combination regimen, in contrast to a continuous dosing schedule.

High baseline *RAS* mutant allele frequency in circulating DNA was associated with a short median OS in placebo-treated patients of the CORRECT clinical trial [62]. Interestingly, our exploratory analysis provided further evidence that patients with high baseline *RAS*MT allele frequency had a significant shorter median OS compared with that seen for patients with low baseline *RAS*MT allele frequency, supporting the previous findings of the phase III CORRECT trial.

Taken together, this study establishes that poor tolerability prevents the combination of binimetinib and crizotinib from being a meaningful therapeutic option, at the tested dosing and schedule, for heavily pre-treated *RAS*MT advanced CRC. Future studies of agents targeting MEK and MET should include alternative and more novel MET mAbs, alternative dosing schedules, and further explore the relationship between genomic alterations and efficacy. Intrinsic or acquired MET amplification in *RAS*MT patients [63] may be a molecular subset where MEK and MET inhibitor combination therapy should be studied further.

## Supplementary Information

Supplementary Material 1.

## Data Availability

Data availability: Raw data for RNA-sequencing has been deposited in SRA database (PRJNA1161993). Requests for use of the individual participant data after publication should be made in writing to the Oncology Clinical Trials Office and will be managed as per contemporaneous applicable data sharing procedures.
