# Supplementary material for: A Phase Ia/b study of MEK1/2 inhibitor binimetinib with MET inhibitor crizotinib in patients with RAS mutant advanced colorectal cancer (MErCuRIC)
Source: BMC Cancer. 2025 Apr 10;25:658. doi: 10.1186/s12885-025-14068-1 (PMC11984268; doi:10.1186/s12885-025-14068-1)
Supplement: Supplementary file 1 — Supplementary Material 1. [file 12885_2025_14068_MOESM1_ESM.pdf]

A.

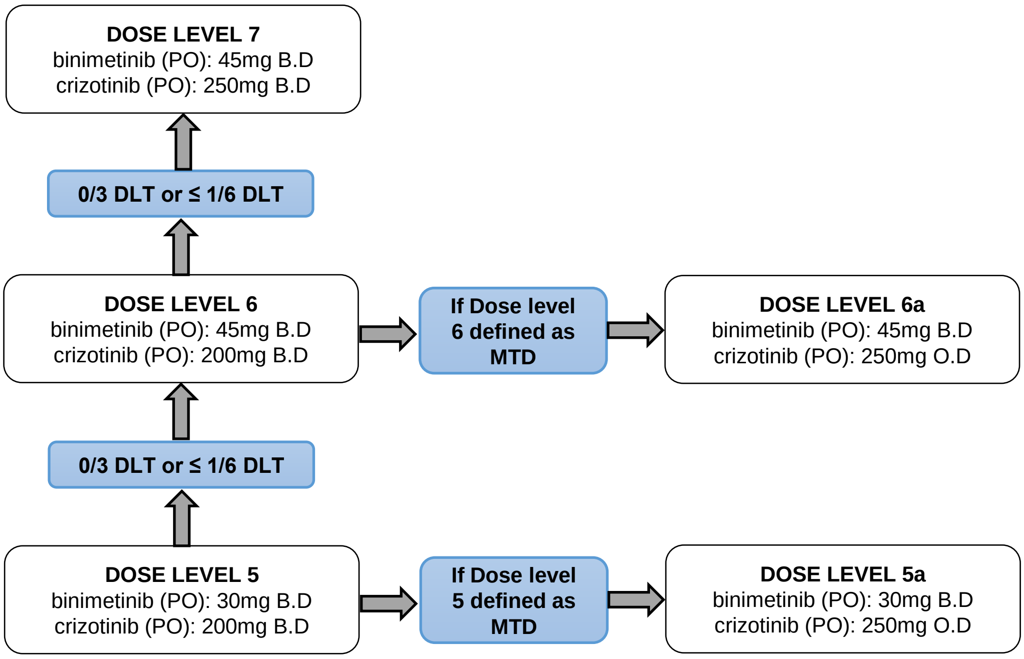

B.

Dose Level Dose escalation	Cohorts Dose escalation	binimetinib		crizotinib
5	7	30mg B.D	Day 1-28	200mg B.D
5*	12	30mg B.D	Day 1-21	200mg B.D
5a*	13	30mg B.D	Day 1-21	250mg O.D

C.

Drug	Cycle 1 -12			
	Week 1	Week 2	Week 3	Week 4
binimetinib	Continuous administration			
	or			
	Days 1-21 every 28 days			
crizotinib	Continuous administration			

D.

***RAS*MT**
Simon optimal: response
p1=0.1
p2=0.25
Power=80%
Alpha=5%

Stage 1: If at least 2 responder in first 22 patients, then continue to stage 2
Stage 2: If at least 7 responders in 40 patients, then continue to phase II

Supplementary figure 1. Schematic overview of the phase Ia study design of crizotinib and binimetinib in patients with advanced solid cancer. A. Trial design and dose escalation schema. Potential exploration of dose levels 5a and 6a if crizotinib 200mg B.D dosing is not well tolerated in the combination treatment. Potential requirement to reduce frequency of the binimetinib dosing schedule from continuous dosing throughout the study period to days 1 to 21 every 28 days dependent on tolerability data. **B.** Dose levels and doses per cohort given in the phase Ia study. **C.** Representation of the treatment schedule. **D.** Sample size calculation for dose expansion phase design.

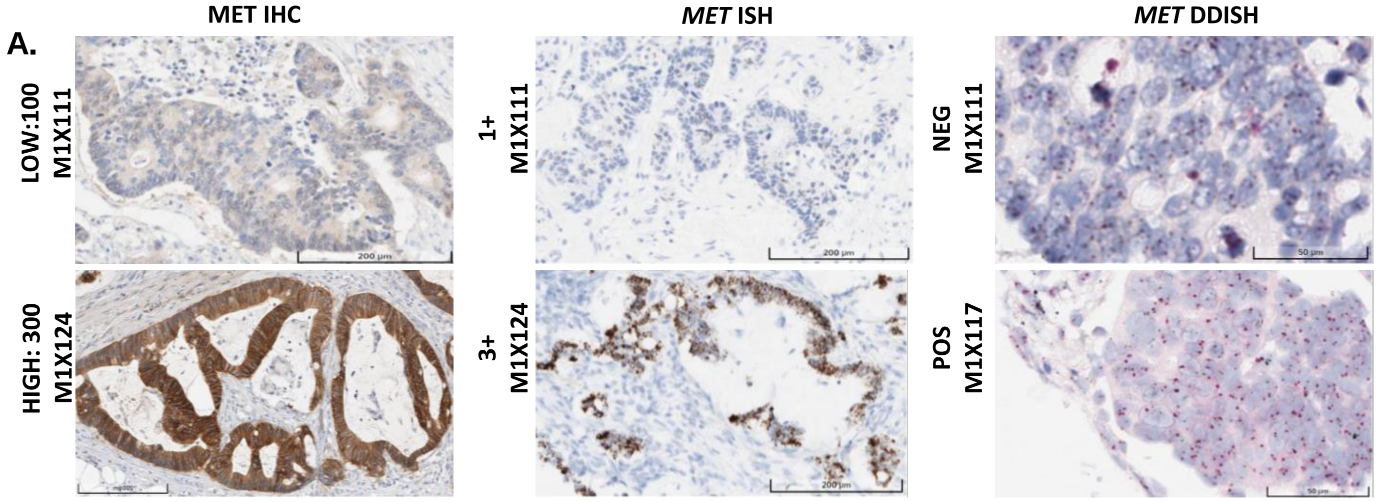

B.

Screening tissue n = 36	No MET Superexpressor not amplified n (%)	MET Superexpressor not amplified n (%)	MET Superexpressor amplified n (%)	No viable tumour cells
All ptn, n (%)	21 (58.3)	9 (25)	1 (2.8)	6 (16.7)
29 RE ptn n, (%)	18 (62.1)	7 (24.1)	1 (3.4)	4 (13.8)

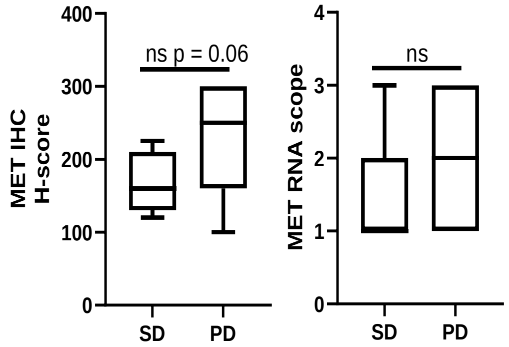

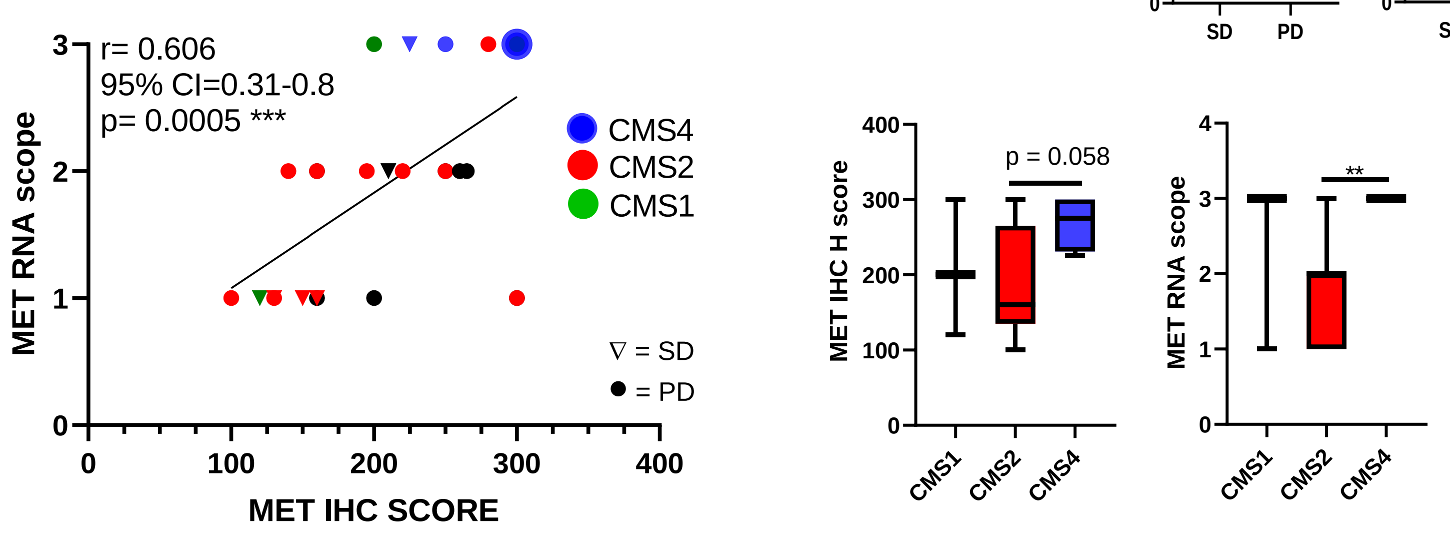

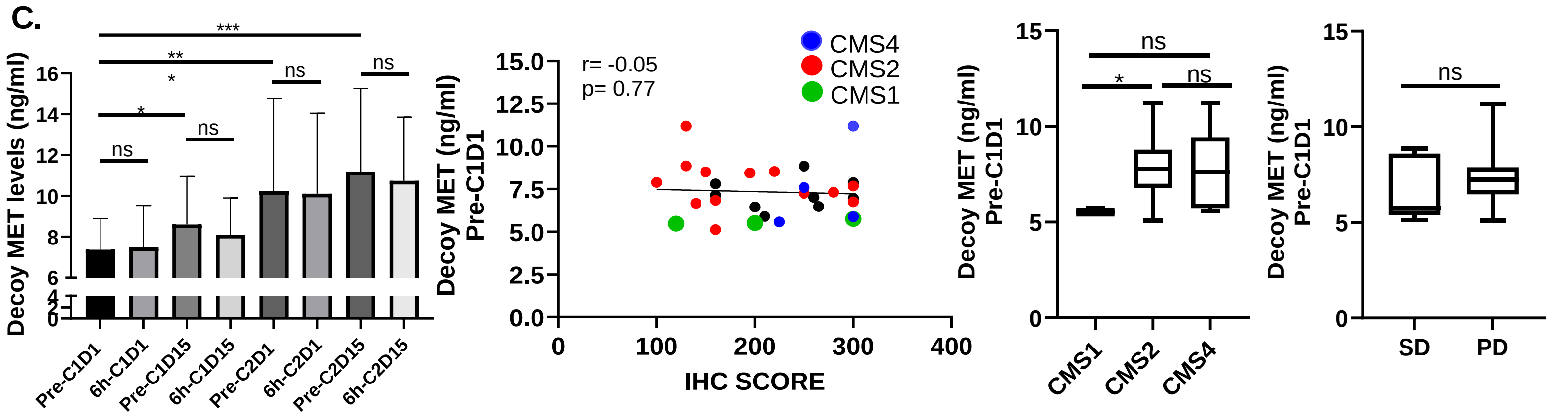

Supplementary figure 2. MET IHC, *MET* ISH and DDISH analysis in the pre-treatment biopsies/archival tumour block from patients in the phase Ib study. A. Representative picture for c-MET IHC staining (LOW: 100 and HIGH: 300), *MET* ISH staining (LOW: +1 and high: +3) and DDISH (negative and positive) in core tumour pre-treatment biopsies/resection specimens. **B. Top left:** MET IHC, *MET* ISH scope and DDISH results in all phase Ib patients (n=36) and in the patient cohort evaluable for response (RE). MET super-expressor (IHC H-score > 180 and *MET* ISH +3). **Top right:** Boxplots showing cMET IHC and *MET* ISH scores in phase Ib patients that obtained stable disease (SD) and progressive disease (PD). **Bottom left:** Pearson's correlation of cMET IHC scores and *MET* ISH scores. **Bottom right:** Boxplots showing MET IHC and *MET* ISH levels in each of CMS groups in pre-treatment biopsies/resection specimens. **C. Left:** Results for human c-MET (soluble) in the plasma samples of patients in dose expansion phase during cycle 1 and cycle 2. Pre-C1D1 = C1D1 pre-treatment, 6h-C1D1 = C1D1 6h post-treatment; Pre-C1D15 = C1D15 pre-treatment, 6h-C1D15 = C1D15 6h post-treatment; Pre-C2D1 = C2D1 pre-treatment, 6h-C2D1 = C2D1 6h post-treatment; Pre-C2D15 = C2D15 pre-treatment, 6h-C2D15 = C2D15 6h post-treatment. **Middle:** Pearson's correlation of baseline C1D1 pre-treatment c-MET soluble plasma levels and MET IHC scores. **Right:** Boxplots showing soluble (Decoy) MET levels in each of CMS groups. Boxplots showing soluble (Decoy) MET levels in phase Ib patients that obtained stable disease (SD) and progressive disease (PD).

A.

gene	exons
AKT1	Exon 2-3-4
CTNNB1	exon2-3
EGFR	exon12-18-19-20-21
ESR1	exon6-10
EZH2	exon16
FGFR2	exon7-9-12-14
FGFR3	exon7-9-14-16
GNA11	exon4-5
GNAQ	exon5
GNAS	exon8-9
HRAS	exon2-3
IDH1	exon4
IDH2	exon4
KIT	exon3-8-11-13-14-15-17
KRAS	exon2-3-4
MAP2K1	exon2-3-6
MAP2K2	exon1-2-6-7

gene	exons
MAPK1	exon3-7
MAPK3	exon3-4-7-8
NFE2L2	exon2
NRAS	exon2-3-4
NTRK1	exon13-14
PDGFRA	exon12-14-18
PIK3CA	exon9-20
POLE	Exon9-13-14-34-1[illegible]
PTEN	exon5-6-7-8
PTPN11	exon3-13
RET	exon2-11-15-16
RNF43	exon2-3-4-5-8
SMAD4	exon3-9-10-11-12
SMO	exon6-9-11
SRC	exon12-14
STK11	exon1-4-6-8
TERT	5'utr + promoter

gene	exons
APC	all isoforms
BRAF	all isoforms
ERBB2	all isoforms
MET	all isoforms + int 13,14
TP53	all isoforms

gene	exons
MLH1	all isoforms
MSH2	all isoforms
MSH6	all isoforms
PMS2	all isoforms+prom
B2M	Exon 1-2

gene	exons
SNP_ID	8101

	bp
MSI marker	1634

B.

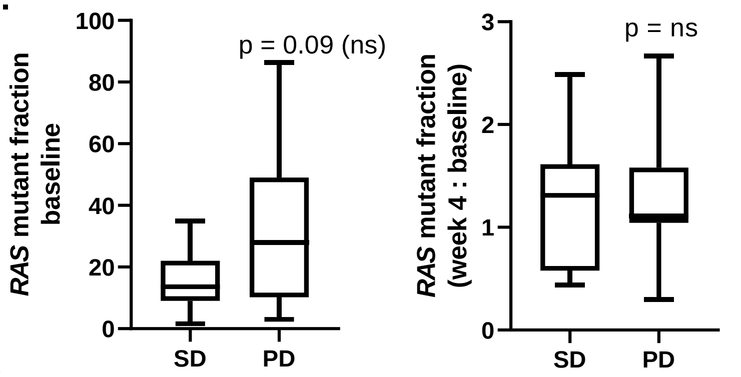

C.

M1X121

Cosmic	Gene	Description	coord	Nchange	AAchange	var_effect	C1D1	EOT	type
6023	KRAS	Kirsten rat sarcoma viral oncogene homolog	chr12:25398285	c.G34A	p.G12S	nonsynonymous	19.1193	47.544	exonic
338	GNAS	GNAS complex locus	chr20:57484420	c.C2530T	p.R844C	nonsynonymous	0.118718	-	exonic
50	TP53	tumor protein p53	chr17:7577117	c.T821G	p.V274G	nonsynonymous	-	0.2028	
5	TP53	tumor protein p53	chr17:7579472	c.C215G	p.P72R	nonsynonymous	39.3658	29.206	exonic
1	RNF43	ring finger protein 43	chr17:56436109	c.G1028A	p.R343H	nonsynonymous	53.7607	56.24	exonic
1	APC	adenomatous polyposis coli	chr5:112175763	c.T4472A	p.F1491Y	nonsynonymous	0.246184	0.9009	exonic
0	RNF43	ring finger protein 43	chr17:56435885	c.C1252A	p.L418M	nonsynonymous	100	99.964	exonic
0	APC	adenomatous polyposis coli	chr5:112176756	c.T5465A	p.V1822D	nonsynonymous	99.9351	99.962	exonic
0	MSH6	mutS homolog 6	chr2:48010488	c.G116A	p.G39E	nonsynonymous	50.4016	49.251	exonic
0	ERBB2	v-erb-b2 avian erythroblastic leukemia viral oncogene homolog 2	chr17:37884037	c.C3508G	p.P1170A	nonsynonymous	43.8137	42.258	exonic
0	ERBB2	v-erb-b2 avian erythroblastic leukemia viral oncogene homolog 2	chr17:37871547	c.C1157A	p.A386D	nonsynonymous	42.4132	44.805	exonic
0	APC	adenomatous polyposis coli	chr5:112173668	c.C2377T	p.Q793*	stopgain	12.6984	23.843	exonic
0	RNF43	ring finger protein 43	chr17:56440961	c.G376T	p.A126S	nonsynonymous	0.193705	-	exonic
0	ESR1	estrogen receptor 1	chr6:152265602	c.G1061T	p.R354M	nonsynonymous	0.182949	-	exonic
0	SMAD4	SMAD family member 4	chr18:48575108	c.G302T	p.W101L	nonsynonymous	-	0.531	exonic
0	BRAF	v-raf murine sarcoma viral oncogene homolog B	chr7:140476803	c.T1603C	p.S535P	nonsynonymous	-	0.2764	exonic
0	APC	adenomatous polyposis coli	chr5:112175760	c.A4469T	p.H1490L	nonsynonymous	-	0.1506	exonic
0	RET	ret proto-oncogene	chr10:43596133	c.C300A	p.S100R	nonsynonymous	-	0.1272	exonic

M1X118

Cosmic	Gene	Description	coord	Nchange	AAchange	var_effect	C1D1	EOT	type
19100	KRAS	Kirsten rat sarcoma viral oncogene homolog	chr12:25398284	c.G35A	p.G12D	nonsynonymous	8.46395	5.90462	exonic
340	TP53	tumor protein p53	chr17:7578212	c.C637G	p.R213G	nonsynonymous	10.6203	9.48454	exonic
27	APC	adenomatous polyposis coli	chr5:112175216	c.G3925T	p.E1309*	stopgain	0.365965	-	exonic
5	TP53	tumor protein p53	chr17:7579472	c.C215G	p.P72R	nonsynonymous	42.1864	45.2775	exonic
2	MSH2	mutS homolog 2	chr2:47643457	c.G965A	p.G322D	nonsynonymous	47.7816	46.0751	exonic
1	MLH1	mutL homolog 1	chr3:37053568	c.A655G	p.I219V	nonsynonymous	52.2571	51.6279	exonic
0	ERBB2	v-erb-b2 avian erythroblastic leukemia viral oncogene homolog 2	chr17:37884037	c.C3508G	p.P1170A	nonsynonymous	100	100	exonic
0	NTRK1	neurotrophic tyrosine kinase, receptor, type 1	chr1:156848918	c.C1810T	p.H604Y	nonsynonymous	100	100	exonic
0	NTRK1	neurotrophic tyrosine kinase, receptor, type 1	chr1:156848946	c.G1838T	p.G613V	nonsynonymous	100	100	exonic
0	APC	adenomatous polyposis coli	chr5:112176756	c.T5465A	p.V1822D	nonsynonymous	99.8895	100	exonic
0	ERBB2	v-erb-b2 avian erythroblastic leukemia viral oncogene homolog 2	chr17:37855834	c.C22A	p.P8T	nonsynonymous	44.898	45.7045	exonic
0	RNF43	ring finger protein 43	chr17:56448297	c.G350A	p.R117H	nonsynonymous	50	47.561	exonic

M1X108

Cosmic	Gene	Description	coord	Nchange	AAchange	var_effect	C1D1	C4D4	type
19100	KRAS	Kirsten rat sarcoma viral oncogene homolog	chr12:25398284	c.G35A	p.G12D	nonsynonymous	0.927703	10.3267	exonic
1057	TP53	tumor protein p53	chr17:7578406	c.G524A	p.R175H	nonsynonymous	1.22378	12.4843	exonic
22	SMAD4	SMAD family member 4	chr18:48591919	c.G1082A	p.R361H	nonsynonymous	0.672948	7.93333	exonic
5	TP53	tumor protein p53	chr17:7579472	c.C215G	p.P72R	nonsynonymous	100	100	exonic
2	MET	met proto-oncogene	chr7:116339642	c.G504T	p.E168D	nonsynonymous	-	0.316456	exonic
0	ERBB2	v-erb-b2 avian erythroblastic leukemia viral oncogene homolog 2	chr17:37884037	c.C3508G	p.P1170A	nonsynonymous	100	100	exonic
2	RET	ret proto-oncogene	chr10:43610119	c.G2071A	p.G691S	nonsynonymous	-	0.140581	exonic
0	RNF43	ring finger protein 43	chr17:56448297	c.G350A	p.R117H	nonsynonymous	100	100	exonic
0	MSH6	mutS homolog 6	chr2:48010488	c.G116A	p.G39E	nonsynonymous	51.167	50.8701	exonic
0	MSH2	mutS homolog 2	chr2:47702191	c.A1787G	p.N596S	nonsynonymous	50.3796	51.8456	exonic
0	APC	adenomatous polyposis coli	chr5:112176756	c.T5465A	p.V1822D	nonsynonymous	48.9388	57.5428	exonic
0	ERBB2	v-erb-b2 avian erythroblastic leukemia viral oncogene homolog 2	chr17:37855834	c.C22A	p.P8T	nonsynonymous	44.1699	42.2403	exonic
0	APC	adenomatous polyposis coli	chr5:112116562	c.C607G	p.Q203E	nonsynonymous	-	0.31506	exonic
0	APC	adenomatous polyposis coli	chr5:112178795	c.G7504A	p.G2502S	nonsynonymous	-	0.243546	exonic
0	EGFR	epidermal growth factor receptor	chr7:55259424	c.T2482G	p.L828V	nonsynonymous	-	0.207147	exonic

Supplementary figure 3. RAS hotspot mutant allele and NGS analysis of liquid biopsies from patients in the phase Ib study. **A.** NGS custom LB panel and regions captured in the custom panel. **B.** Baseline RAS mutation levels in cfDNA (**left**) and percentage change in RAS mutation levels in cfDNA (week 4 vs baseline) (**right**) for patients achieving stable disease (SD) or progressive disease (PD). *P* values represent SD vs PD by 2-tailed *t* test. **C**. Clinical report of mutations at baseline and end of treatment timepoints for M1X108, M1X118 and M1X121: the fractional abundances at two timepoints, the number of occurrences in COSMIC database, description of the mutated gene, genomic coordinates on human genome v37, nucleotide change, amino acid change and genomic region type were reported.

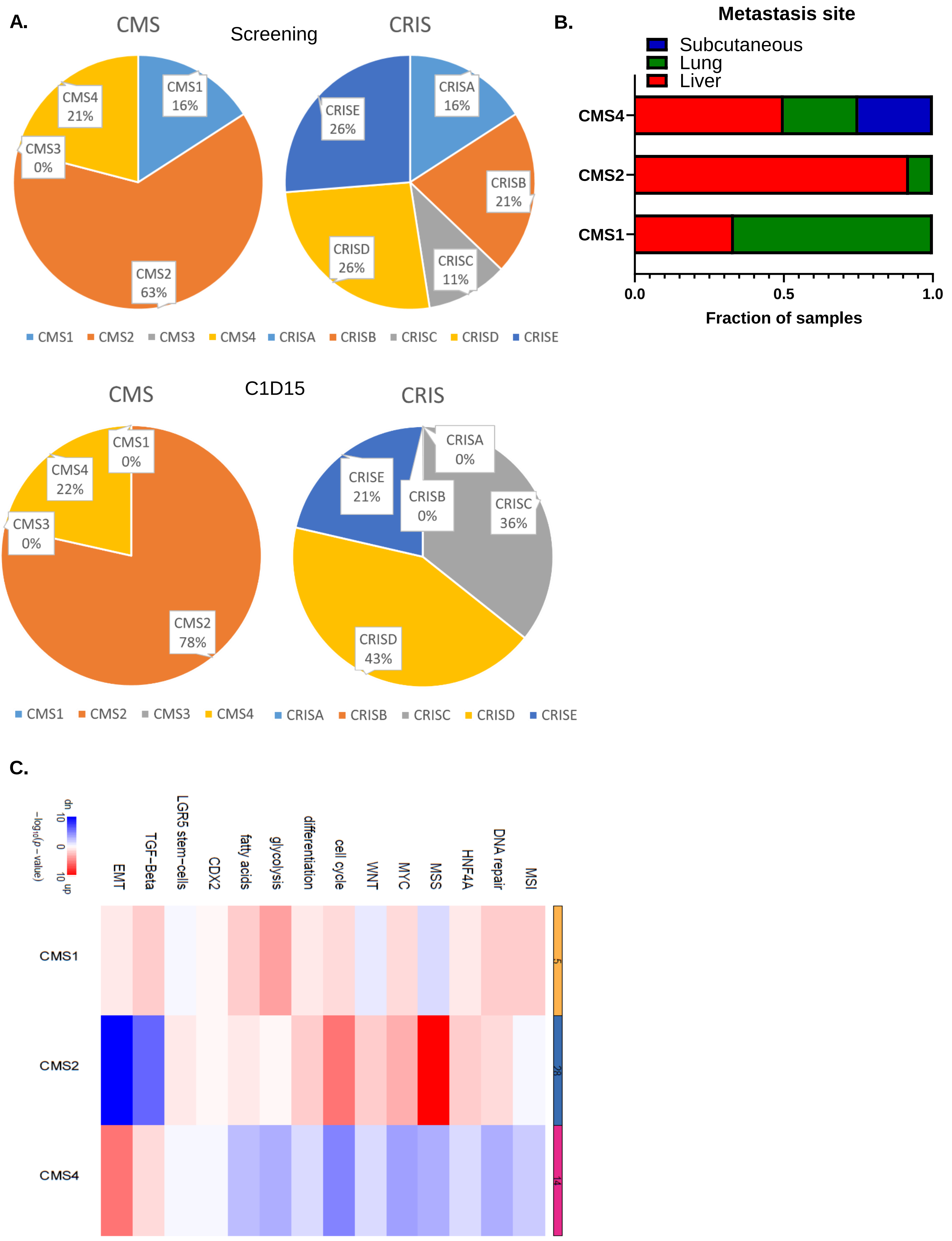
A.
CMS
Screening
CRIS
CMS1 16%
CMS4 21%
CMS3 0%
CMS2 63%
CRISA 16%
CRISE 26%
CRISB 21%
CRISD 26%
CRISC 11%
CMS1 CMS2 CMS3 CMS4 CRISA CRISB CRISC CRISD CRISE
CMS
C1D15
CRIS
CMS1 0%
CMS4 22%
CMS3 0%
CMS2 78%
CRISA 0%
CRISE 21%
CRISB 0%
CRISC 36%
CRISD 43%
CMS1 CMS2 CMS3 CMS4 CRISA CRISB CRISC CRISD CRISE
B.
Metastasis site
Subcutaneous
Lung
Liver
CMS4
CMS2
CMS1
0.0
0.5
1.0
Fraction of samples
C.
EMT
TGF−Beta
LGR5 stem−cells
CDX2
fatty acids
glycolysis
differentiation
cell cycle
WNT
MYC
MSS
HNF4A
DNA repair
MSI
CMS1
CMS2
CMS4
5
28
14

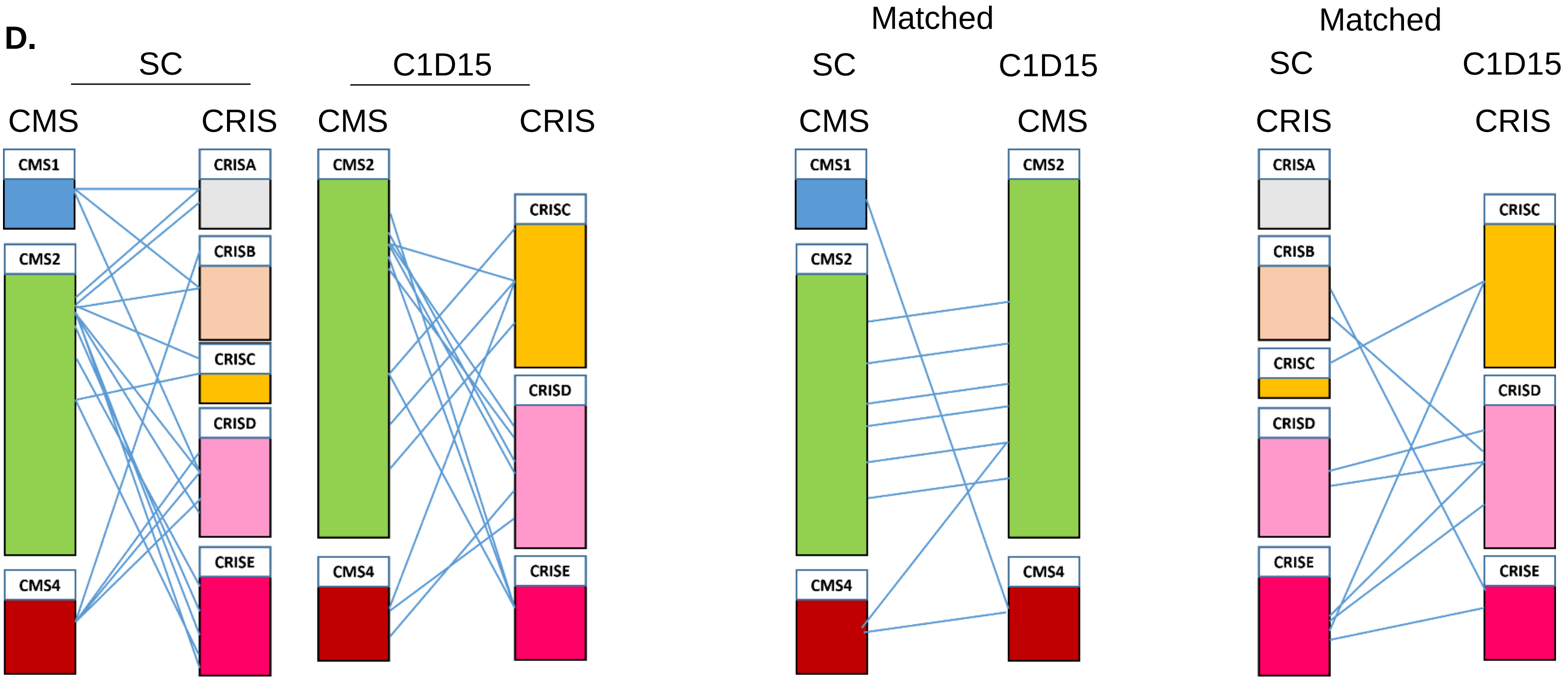

Supplementary figure 4. CMS and CRIS classification of the screening (SC) and C1D15 tumour samples. A. Prevalence of CMS and CRIS groups in the screening and C1D15 metastatic CRC biopsies. **B.** Distribution of metastatic dissemination sites for each CMS group in the MErCuRIC cohort (screening biopsies). **C.** Heatmap showing pathway up- and downregulated in the pre-treatment metastatic biopsies, classified as CMS1, CMS2 and CMS4 tumours. **D.** Caleydo view of correspondences between CMS subtypes and CRIS classes in the phase Ib clinical samples. SC screening samples; C1D15 tumour samples.

• Neutropenia Grade 4 for ≥5 days duration
• Febrile neutropenia (without clinically or microbiologically documented infection) with an absolute neutrophil count <1000/mm^3 and a single temperature of >38.3˚C or a sustained temperature of ≥38˚C for more than one hour.
• Infection (documented clinically or microbiologically) with Grade 3 or 4 neutropenia (ANC <1.0 x 10^9/L)
• Thrombocytopenia Grade 4:<br>a) for ≥5 days duration, or<br>b) associated with active bleeding, or<br>c) requiring platelet transfusion.
• Symptomatic Grade 3 CPK elevation or Grade 4 asymptomatic CPK elevation
• Grade 3 or 4 toxicity to organs other than the bone marrow but including Grade 3 and Grade 4 biochemical AEs EXCLUDING:<br>Grade 3 nausea in patients who have not received optimal treatment with anti-emetics<br>Grade 3 or 4 vomiting in patients who have not received optimal treatment with anti-emetics<br>Grade 3 or 4 diarrhoea in patients who have not received optimal treatment with anti-diarrhoeals and Grade 2 diarrhoea for more than 7 days in patients who have received optimal treatment with anti-diarrhoeals.<br>Grade 3 fatigue, unless there is an increase by at least two grades from baseline
• AE with a fatal outcome

Supplementary Table 1. Definition of DLT in the phase I dose escalation. DLTs identified during the first cycle informed the decision to dose escalate through the increasing dose levels. A DLT was defined as an almost certainly or probably drug-related adverse event to crizotinib and/or binimetinib.

	Dose escalation				Dose expansion
**No. of patients affected**<br>binimetinib<br><br>crizotinib<br>n, (%)	Cohort 7<br>30mg B.D<br>days 1-28<br>200mg B.D<br>(n=8)	Cohort 12<br>30mg B.D<br>days 1-21<br>200mg B.D<br>(n=5)	Cohort 13<br>30mg B.D<br>days 1-21<br>250mg O.D<br>(n=7)	Total<br>(n=20)	Total<br>(n=36)
**SAE**					
**Constipation**	1 (12.5)	0 (0)	0 (0)	1 (5)	2 (5.56)
**Diarrhoea**	0 (0)	1 (20)	0 (0)	1 (5)	1 (2.78)
**Dyspnoea**	1 (12.5)	0 (0)	0 (0)	1 (5)	1 (2.78)
**Nausea**	0 (0)	0 (0)	0 (0)	0 (0)	1 (2.78)
**Vomiting**	0 (0)	0 (0)	0 (0)	0 (0)	1 (2.78)
**Decreased LV Ejection fraction/cardiac failure**	1 (12.5)	0 (0)	0 (0)	1 (5)	1 (2.78)
**Pericarditis**	0 (0)	0 (0)	0 (0)	0 (0)	1 (2.78)
**Ascites**	1 (12.5)	0 (0)	0 (0)	1 (5)	0 (0)
**Pleural effusion**	0 (0)	0 (0)	0 (0)	0 (0)	1 (2.78)
**Pulmonary infection**	1 (12.5)	1 (20)	1 (14.3)	3 (15)	2 (5.56)
**Pain**	0 (0)	0 (0)	1 (14.3)	1 (5)	2 (5.56)
**Pneumonitis**	1 (12.5)	0 (0)	0 (0)	1 (5)	0 (0)
**Postural hypotension**	0 (0)	0 (0)	1 (14.3)	1 (5)	0 (0)
**Skin infection (PICC line related)**	1 (12.5)	0 (0)	0 (0)	1 (5)	0 (0)
**Thromboembolic event**	0 (0)	0 (0)	1 (14.3)	1 (5)	2 (5.56)
**Mucositis**	0 (0)	0 (0)	0 (0)	0 (0)	1 (2.78)
**Retinopathy**	0 (0)	0 (0)	0 (0)	0 (0)	1 (2.78)
**ALT and/or ALT increase**	0 (0)	0 (0)	0 (0)	0 (0)	2 (5.56)
**Brain metastases**	0 (0)	0 (0)	1 (14.3)	1 (5)	0 (0)
**Hepatic Haemorrhage**	0 (0)	0 (0)	0 (0)	0 (0)	1 (2.78)
**Vertigo**	0 (0)	0 (0)	0 (0)	0 (0)	1 (2.78)
**Dehydration**	0 (0)	0 (0)	0 (0)	0 (0)	1 (2.78)
**Sensory neuropathy**	0 (0)	0 (0)	0 (0)	0 (0)	1 (2.78)
**DLT**	**(n=6)**	**(n=5)**	**(n=6)**	**(n=17)**	
**CPK increase**	1 (16.7)	1 (20)	0	2 (11.7)	
**ALT and or ALT increase**	1 (16.7)	1 (20)	0	2 (11.7)	
**Fatigue**	0	0	1 (16.7)	1 (5.9)	

Supplementary Table 2. Serious Adverse Events (SAEs) and Dose Limiting Toxicities (DLTs) experienced by patients in Cohort 7, 12 and 13. Serious Adverse Events (SAEs) in dose expansion phase.

**Dose escalation**<br>**No. of patients AE affected**	**Cohort 7 (n=8)**		**Cohort 12 (n=5)**		**Cohort 13 (n=7)**	
	Any grade n, (%)	Grade ≥ 3 n, (%)	Any grade n, (%)	Grade ≥ 3 n, (%)	Any grade n, (%)	Grade ≥ 3 n, (%)
**NON-HAEMATOLOGICAL AND NON-BIOCHEMICAL**						
Rash	8 (100)	0 (0)	4 (80)	0 (0)	7 (100)	0 (0)
Pruritus	1 (12.5)	0 (0)	0 (0)	0 (0)	0 (0)	0 (0)
Skin Dryness	0 (0)	0 (0)	1 (20)	0 (0)	0 (0)	0 (0)
Purpura	0 (0)	0 (0)	0 (0)	0 (0)	1 (14.3)	0 (0)
Nausea	3 (37.5)	0 (0)	2 (40)	0 (0)	4 (57.1)	0 (0)
Dyspepsia	0 (0)	0 (0)	1 (20)	0 (0)	0 (0)	0 (0)
Dry mouth	1(12.5)	0 (0)	0 (0)	0 (0)	1 (14)	0 (0)
Mucositis (mouth)	0 (0)	0 (0)	0 (0)	0 (0)	2 (28.6)	0 (0)
Vomiting	1 (12.5)	0 (0)	0 (0)	0 (0)	3 (42.9)	0 (0)
Anorexia	2 (25)	0 (0)	1 (20)	0 (0)	0 (0)	0 (0)
Diarrhoea	7 (87.5)	0 (0)	3 (60)	0 (0)	3 (42.9)	0 (0)
Constipation	1 (12.5)	0 (0)	0 (0)	0 (0)	0 (0)	0 (0)
Abdominal cramps	0 (0)	0 (0)	1 (20)	0 (0)	0 (0)	0 (0)
Fatigue	5 (62.5)	0 (0)	3 (60)	0 (0)	6 (85.7)	1 (14.3)
Oedema	4 (50)	0 (0)	1 (20)	0 (0)	3 (42.9)	1 (14.3)
Arthralgia/myalgia	1 (12.5)	0 (0)	2 (40)	0 (0)	1 (14.3)	0 (0)
Dizziness	0 (0)	0 (0)	0 (0)	0 (0)	2 (28.6)	0 (0)
LV Ejection fraction ↓	1 (12.5)	1 (12.5)	0 (0)	0 (0)	1 (14.3)	0 (0)
Pleural effusion	1 (12.5)	1 (12.5)	0 (0)	0 (0)	0 (0)	0 (0)
Pericardial effusion	1 (12.5)	0 (0)	0 (0)	0 (0)	0 (0)	0 (0)
Ventricular arythmia	0 (0)	0 (0)	1 (20)	0 (0)	0 (0)	0 (0)
Retrosternal chest pain	0 (0)	0 (0)	1 (20)	0 (0)	0 (0)	0 (0)
Dyspnoea	2 (25)	1 (12.5)	0 (0)	0 (0)	0 (0)	0 (0)
Postural Hypotension	0 (0)	0 (0)	0 (0)	0 (0)	1 (14.3)	1 (14.3)
Blurred vision	3 (37.5)	0 (0)	0 (0)	0 (0)	1 (14.3)	0 (0)
Eye disorder (Blepharitis)	1 (12.5)	0 (0)	1 (20)	0 (0)	0 (0)	0 (0)
Dryness of the conjunctiva	0 (0)	0 (0)	1 (20)	0 (0)	0 (0)	0 (0)
Arterial hypertension	1 (12.5)	0 (0)	0 (0)	0 (0)	0 (0)	0 (0)
Peripheral neuropathy	2 (25)	0 (0)	0 (0)	0 (0)	2 (28.6)	0 (0)
Paraesthesia	0 (0)	0 (0)	0 (0)	0 (0)	1 (14.3)	0 (0)
Pneumonia	1 (12.5)	1 (12.5)	0 (0)	0 (0)	0 (0)	0 (0)
Cough	2 (25)	0 (0)	0 (0)	0 (0)	0 (0)	0 (0)
Wheezing	0 (0)	0 (0)	0 (0)	0 (0)	1 (14.3)	0 (0)
Fever	1 (12.5)	0 (0)	0 (0)	0 (0)	0 (0)	0 (0)
Alopecia	0 (0)	0 (0)	1 (20)	0 (0)	0 (0)	0 (0)
Weight gain	0 (0)	0 (0)	0 (0)	0 (0)	1 (14.3)	0 (0)
**HAEMATOLOGICAL AND BIOCHEMICAL**						
Anemia	1 (12.5)	0 (0)	0 (0)	0 (0)	0 (0)	0 (0)
Neutropenia	0 (0)	0 (0)	1 (20)	0 (0)	0 (0)	0 (0)
Thrombocytopenia	0 (0)	0 (0)	1 (20)	0 (0)	0 (0)	0 (0)
Hyponatriemia	0 (0)	0 (0)	1 (20)	0 (0)	0 (0)	0 (0)
Hypopotassemia	0 (0)	0 (0)	1 (20)	0 (0)	0 (0)	0 (0)
Hypoalbuminaemia	0 (0)	0 (0)	1 (20)	0 (0)	1 (14.3)	0 (0)
Hyperglycaemia	0 (0)	0 (0)	0 (0)	0 (0)	1 (14.3)	0 (0)
ALT and/or AST increase	2 (25)	1 (12.5)	3 (60)	1 (20)	0 (0)	0 (0)
CPK increase	4 (50)	2 (25)	2 (40)	1 (20)	2 (28.6%)	0 (0)

Supplementary Table 3. Treatment-related adverse events (AE) experienced by the 8 patients in cohort 7, 5 patients in cohort 12 and the 7 patients in Cohort 13, treated at the MTD, who started treatment, by CTCAE grade.

Table 4

	Dose escalation phase				Dose expansion phase
**Cohort**<br>binimetinib<br>crizotinib	**Cohort 7**<br>30mg B.D 28d<br>200mg B.D<br>n = 7	**Cohort 12**<br>30mg B.D 21d<br>200mg B.D<br>n = 5	**Cohort 13**<br>30mg B.D 21d<br>250mg O.D<br>n = 6	p-value	**Dose expansion phase**<br>30mg B.D 21d<br>250mg O.D<br>n = 26
**crizotinib**					
**Cycle 1, Day 21**					
C_{max}, ng/ml (SD)	273 (118)	250 (145)	186 (36.8)	0.3646	197 (112)
C_{min}, ng/ml (SD)	205 (78.0)	181 (136)	114 (43.1)	0.2077	149 (85.3)
T_{max}, h (SD)	4.03 (1.62)	2.19 (1.11)	3.01 (2.01)	0.189	4.03 (1.89)
$AUC_{0\text{-}10h}$, ng*h/ml (SD)	2481 (980)	2119 (1341)	1467 (410)	0.1948	1647 (907)
$C_{min\ 24h}$, ng/ml (SD)	ND	ND	72.6 (17.1)		109 (71.4)
$AUC_{0\text{-}24h}$, ng*h/ml (SD)	ND	ND	2529 (380)		3479 (2083)
**binimetinib**					
**Cycle 1, Day 21**					
C_{max}, ng/ml (SD)	265 (83.4)	386 (189)	320 (136)	0.3406	357 (171)
C_{min}, ng/ml (SD)	77.1 (40.3)	85.8 (51.9)	64.0 (28.9)	0.6729	103 (58.5)
T_{max}, h (SD)	2.26 (2.14)	1.75 (0.5)	1.51 (0.54)	0.6282	2.55 (1.5)
$AUC_{0\text{-}10h}$ ng*h/ml (SD)	1604 (554)	1780 (706)	1402 (666)	0.6235	2129 (1152)
**AR0042603**					
**Cycle 1, Day 21**					
C_{max}, ng/ml (SD)	25.1 (15.1)	29.5 (8.62)	23.4 (8.04)	0.6764	22.7 (16.0)
C_{min}, ng/ml (SD)	10.4 (2.66)	10.2 (2.47)	6.95 (1.6)	0.03 (*)	12.1 (4.8)
T_{max}, h (SD)	3.03 (1.97)	1.75 (0.5)	1.84 (1.16)	0.2398	3.15 (1.99)
$AUC_{0\text{-}10h}$ ng*h/ml (SD)	203 (112)	183 (24.8)	121 (28.9)	0.1577	194 (85.2)

Supplementary Table 4. Pharmacokinetic parameters during dose escalation for binimetinib with crizotinib for each cohort and dose expansion phase as measured during cycle 1. C_{max} = observed maximum concentration post-dose; T_{max} = time to reach maximum concentration in hours; $AUC_{0\text{-}10h}$ = area under concentration-time curve to the last data point at 10h; $AUC_{0\text{-}24h}$ = area under concentration-time curve to the last data point at 24h. ND = not determined. AR0042603 = a metabolite of binimetinib. One-way Anova was used to determine differences between different cohorts.

ID M1X	Tissue	Liquid biopsy baseline (% fractional abundance)
101	KRAS mutant (NS)	KRAS G12C (28,00)
102	KRAS codon 12	KRAS G12V (57,57)
103	KRAS c. 38G>A; pGly13Asp; G13D	KRAS G13D (34,17)
104	KRAS c.35G>A; pGly12Asp; G12D	KRAS G12D (3,87)
105	KRAS c.35G>A; pGly12Asp; G12D	KRAS G12D (10,33)
106	KRAS c.34G>T; pGly12Cys; G12C	KRAS G12C (42,65)
107	KRAS c.38G>A; pGly13Asp; G13D	KRAS G13D (32,15)
108	KRAS c.35G>A; p.Gly12Asp; G12D	KRAS G12D (1,60)
109	KRAS c.35G>T; p.Gly12Val; G12V	KRAS G12V (25,65)
110	KRAS c.38G>A; pGly13Asp; G13D	KRAS G13D (negative)
111	KRAS c.35G>A; pGly12Asp; G12D	KRAS G12D (86,33)
112	KRAS c.38G>A; pGly13Asp; G13D	KRAS G13D (41,05)
113	KRAS c.351A>T; p.Lys117Asn; K117N	negative
114	KRAS c.35G>T; p.Gly12Val; G12V	KRAS G12V (32,00)
115	KRAS c.34G>T; pGly12Cys; G12C	KRAS G12C (3,05)
116	KRAS c.35G>A; pGly12Asp; G12D	KRAS G12D (3,05)
117	KRAS c.38G>A; pGly13Asp; G13D	KRAS G13D (21,3)
118	KRAS c.35G>A; pGly12Asp; G12D	KRAS G12D (10,5)
119	KRAS c.35G>C; pGly12Ala; G12A	KRAS G12A (39,75)
120	NRAS mutant (NS)	NRAS Q61L (5,65)
121	KRAS c.34G>A; pGly12Ser; G12S	KRAS G12S (22,00)
122	KRAS c.35G>A; pGly12Asp; G12D	KRAS G12D (44,00)
123	KRAS c.38G>A; pGly13Asp	ND
124	KRAS c.35G>C; pGly12Ala; G12A	KRAS G12A (5,60)
125	KRAS c.436G>A; p.Ala146Thr; A146T	KRAS A146T (15,00)
126	KRAS c.35G>A; pGly12Asp; G12D	KRAS G12D (13,6)
127	KRAS c.35G>T; p.Gly12Val; G12V	KRAS G12V (34,9)
128	KRAS c.35G>T; p.Gly12Val; G12V	KRAS G12V (10,2)
129	KRAS c.35G>T; p.Gly12Val; G12V	KRAS G12V (71,75)
130	KRAS c.35G>A; pGly12Asp; G12D	KRAS G12D (9,00)
131	KRAS c.38G>A; pGly13Asp; G13D	KRAS G13D (39,9)
132	KRAS c.38G>A; pGly13Asp; G13D	ND
133	KRAS c.38G>A; pGly13Asp; G13D	KRAS G13D (13,95)
134	NRAS exon 3	NRAS Q61K (37,00)
135	KRAS c.38G>A; pGly13Asp; G13D	KRAS G13D (65,9)
136	KRAS c.35G>T; p.Gly12Val; G12V	KRAS G12V (13,57)

Supplementary Table 5. RAS Mutations detected in tissue-based analysis as compared with liquid biopsy in the 36 patients of dose expansion phase. ND: not determined.
